# Supplementary material for: Towards complete and error-free genome assemblies of all vertebrate species
Source: Nature. 2021 Apr 28;592(7856):737–46. doi: 10.1038/s41586-021-03451-0 (PMC8081667; doi:10.1038/s41586-021-03451-0)
Supplement: Supplementary file 1 — This file contains Supplementary text, Supplementary Notes 1-7, Supplementary Figures 1-6 and Supplementary references. [file 41586_2021_3451_MOESM1_ESM.pdf]

nature portfolio

https://doi.org/10.1038/s41586-021-03451-0

Supplementary information

Towards complete and error-free genome assemblies of all vertebrate species

In the format provided by the authors and unedited

Towards complete and error-free genome assemblies of all vertebrate species

Rhie *et al.*

Supplementary Notes
Supplementary Methods

Table of Contents

Supplementary Note 1: Improvements to existing methods 4
- Haplotype phasing 4
- Optical mapping 4
- Comparisons of Hi-C data types and scaffolding 5
- Gap filling 6
- Polishing 6
- Comparative iterative scaffolding 6

Supplementary Note 2: Species and alternative assemblies 7
Supplementary Note 3: Resolving missing genomic regions 9
Supplementary Note 4: Assessing overall assembly quality 10
Supplementary Note 5: The Vertebrate Genomes Project 15
Supplementary Note 6: Current advances in technologies 17
Supplementary Note 7: Assembly pipeline using Docker 17
Supplementary Methods 18
- Quality control and contamination screening 18
- Binning 10XG linked reads and Hi-C reads 19
- ENSEMBL annotation pipeline 19
- NCBI annotation pipeline 20
- Chromosome size estimate from karyotype imaging 21
- Weighted read length distributions 23
- Collapsed repeat calculations 25
- BUSCO 25
- Mis-joins and missed-joins in assemblies 26
- Quantifying false duplications with *k-mers* 28
- Reliable blocks 29
  - Gaps in the reference 30
  - CLR coverage 30
  - Linked read coverage 30
  - Optical map raw molecule (bnx) coverage 31
  - Hi-C interaction coverage 32
  - Merging supportive regions 32
- Telomere motifs 34
- Base pair accuracy (QV) estimate 35
  - Mapping based approach 35
  - K-mer based approach 36
- RNA-seq and ATAC-seq Mappability 37
- False gene annotation in previous assemblies 38
- GC-content and missing sequences in prior assemblies 38
- Chromosome evolution analyses 39

Supplementary References 41

Supplementary Note 1: Improvements to existing methods

This supplementary note contains additional findings that we believe will be useful for the genomics community. Some of the results were generated during evaluations involving technology engineers and bioinformaticians from genomics companies, including several co-authors, with whom we actively exchanged information on what we discovered using the genomic richness of the species in this study.

Haplotype phasing

For diploid assembly, we refer to the more continuous pseudo-haplotype assembly as the 'primary' and the other as the 'alternate'; we used the primary contigs for scaffolding. Most of the contigging and scaffolding tools we used were originally designed to handle a haploid representation of the genome. As shown in this study, this design can result in errors in and around heterozygous alleles. We found that such errors introduced from haplotype differences early in the process propagate to later stages, and are not easily corrected. The earlier the haplotypes are sorted in the assembly pipeline, the higher the assembly quality metrics especially for highly heterozygous genomes, as seen with both haplotypes of the female zebra finch assembly in the following order of increasing metric quality: 1) Collapsing first and phasing afterwards (e.g. FALCON-Unzip[1]); 2) Collapsing first and phasing afterwards using Hi-C (e.g. FALCON-Phase[2]); and 3) Phasing reads before assembling into contigs (e.g. TrioCanu[3]).

During curation, we found a pattern of excessive breaks flanking heterozygous sites, which contributed to false duplications in the primary assembly. We traced the source and found FALCON often unnecessarily broke the contigs at the branching point between runs of homozygosity and pairs of heterozygous alleles, reducing the continuity of the assembly. This case is illustrated in types 3 and 4 in **Extended Data Figure 5a**, leaving many homotype duplications at contig boundaries as described in **Extended Data Figure 5b** (left). In response, PacBio fixed the problem in an updated software release (smrtanalysis upgrade, April 2018), which doubled to tripled the contig N50 sizes on a number of genomes.

Haplotypes are essentially a chromosome-scale genomic repeat, and so new methods developed for repeat separation should also help the haplotype assembly problem. The fundamental challenge is distinguishing true genomic variants from errors in the sequencing data, and paralogs from orthologs, and then linking those solutions across the length of full chromosomes. There is a need for improved tools that can better model the diploid (or polyploid) architecture of the genome by integrating long-range evidence from multiple sources, across large repeats, while still preserving haplotype specific variation in the genome.

Optical mapping

Bionano Genomics used our Anna's hummingbird and Kakapo samples to help develop and test their 2-enzyme nicking (BspQI and BssSI) and 1-enzyme non-nicking (DLE-1) approaches for hybrid scaffolding[4]. In early 2015, we found that using two sets of nicking enzyme maps together resulted in better scaffolding continuity compared to using only one (data not shown). This was because the two enzymes compensate each other and eliminate scaffold breaks. Later in 2017, in the development of DLE-1, we found the molecule sizes were superior when avoiding unintentional cutting of genomic DNA at label sites. When applying them to the same FALCON-Unzip primary contigs, we confirmed the scaffold NG50s were higher in the DLE-1 only approach compared to the 2-enzyme hybrid approach (**Supplementary Table 3**). Therefore, we decided to move forward with the latest DLE-1 technology whenever possible.

Comparisons of Hi-C data types and scaffolding

As mentioned in the main text, we tested Hi-C and Chicago libraries on the same Anna's hummingbird sample from three sources (Dovetail Genomics Hi-C v1 and Chicago v1, Phase Genomics v1, and Arima Genomics v1) with versions developed as of mid-2016. We mapped back the paired Hi-C reads using Juicer[5] to the previous reference hummingbird assembly generated with short reads[6], and evaluated interaction size distribution, duplication rate, and genome coverage. We caution that this mapping depends on the structural and base call accuracy of the prior assembly, but all Hi-C datasets were at least compared to the same assembly. We found that insert size (linking distance) differed: Arima > Phase > Dovetail Hi-C > Dovetail Chicago. Dovetail Hi-C tended to have more paired end reads without an insert, which with our feedback they fixed in an upgraded chemistry at the time. Phase Genomics Hi-C had more PCR duplicates, which were possible to screen out. Arima had the highest per base coverage for phasing (**Supplementary Fig. 1**), presumably due to using two enzymes instead of one at the time, but the overall genome assembly was not distinguishable between the data sets. Based on these analyses, we chose to use Arima Hi-C v1 to generate most of the assemblies on other species for this study. We note, though, that each company has continued to make improvements, and thus choice of Hi-C data type will need continued evaluation.

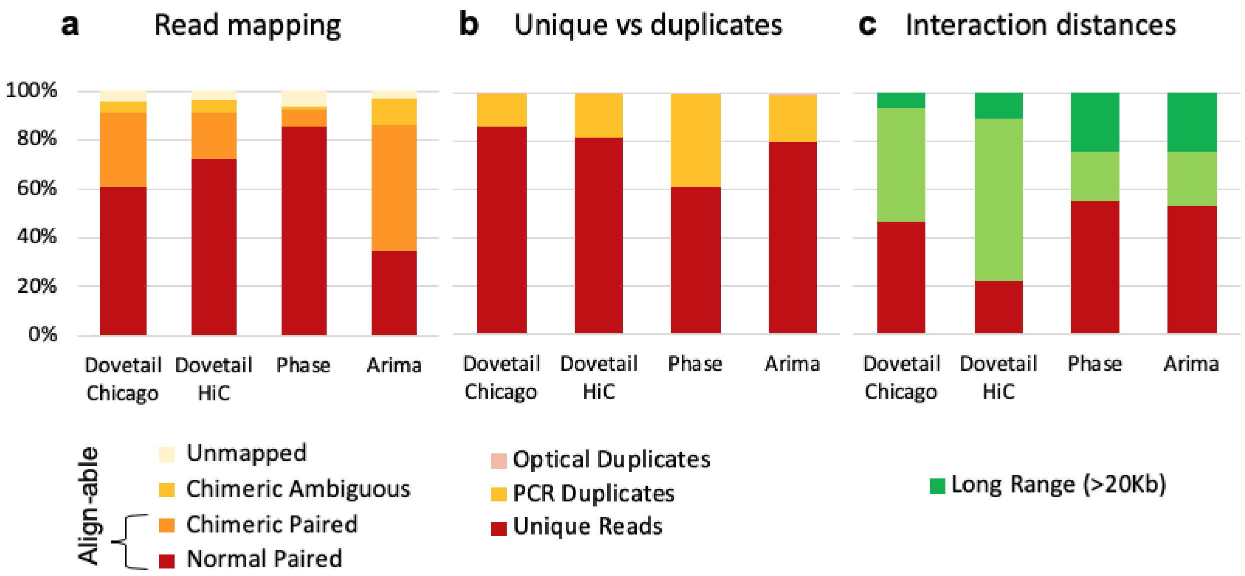

Supplementary Fig. 1 | Read mapping statistics and interaction distance of three Hi-C platforms benchmarked on Anna's hummingbird assembly. a, Read % mapped back to the reference; the higher the alignable portion of the reads mapped the more useful for assembly. **b**, The higher the proportion of unique mapped reads the more useful for assembly; duplicates do not add new information. **c**, The higher the proportion of long range interactions distances between Hi-C read pairs, greater than the long read lengths (~20 kbp for CLR) and spanning contigs, the more useful for chromosomal scale scaffolding. These data sets are from v1 chemistries from each company (Dovetail Genomics, Phase Genomics, and Arima Genomics) used on the samples from the same Anna's hummingbird, mapped back to our newly generated FALCON-Unzip contigs. Each company has made improvements since, including based on the results of this figure and associated data that we provided.

We tested different Hi-C scaffolding algorithms (Phase Genomics Proximo Hi-C; Dovetail Chicago HiRise; 3D-DNA[7], and SALSA[8]). We found that with software versions used at the time, SALSA 2.0 resulted in the highest NG50 metrics with apparent over-scaffolding when tested on the hummingbird Hi-C data sets. With feedback from curation of the hummingbird and other assemblies, we also improved SALSA to SALSA2.2, with a feature that breaks mis-joins introduced in prior scaffolding rounds with support from

Hi-C interactions. These changes simplified and streamlined downstream visualization of the assemblies. As with the Hi-C data types, developers of these algorithms continue to make improvements, including with our iterative feedback, and may thus work differently than the versions we tested.

Gap filling

We attempted to use PBJelly[9] on the hummingbird, a tool developed using a previous G10K supported assembly of the Assemblathon 2.0 budgerigar genome[10] and other genomes to fill in gaps between contigs in scaffolds with PacBio CLR reads. However, we found during evaluation that in addition to properly filling gaps, PBJelly introduced many base call errors in the gap-filled consensus when using default options. The default scaffolding function also introduced mis-joins. Subsequently, we tested Arrow (smrtanalysis 5.1.0.26412 version) on these same assemblies, a consensus base caller that also has a gap filling function, developed by Pacific BioSciences (https://github.com/PacificBiosciences/GenomicConsensus)[11]. Arrow was more conservative and had better consensus quality of the filled gaps (**Supplementary Table 4**). With this result, we replaced PBJelly with Arrow in our initial pipeline.

Polishing

We initially attempted to use Pilon[12] for Illumina SR polishing, and found the best combination of polishing to get the highest QV with minimal steps (**Supplementary Table 5**). However, Pilon was memory intensive to run on large genomes. Thus, we switched to FreeBayes[13] and bcftools[14] for variant calling and consensus generation. During our initial FreeBayes polishing, we encountered regions with excessive coverage (>4000x). We worked with the original author of FreeBayes (E.G. of this study) and found it was hanging on low complexity regions or regions with excessively high coverage. The low complexity issue was fixed by changing the coding in the entropy calculation steps; the excessive coverage issue from loading reads was fixed by setting an upper limit of the coverage (release v1.3.1). These fixes also resulted in optimized memory usage and speed. In all cases of polishing, the reads were mapped to both the primary and alternate haplotype assemblies and the better of the two mappings selected as the primary to avoid reverting haplotype-specific variants.

Comparative iterative scaffolding

When using Hi-C scaffolding alone, we found there were a higher number of inversion errors for the smaller contigs as reported in Ghurye et al[8] (**Supplementary Table 3**), which can be difficult to orient correctly using long-range data alone. Optical mapping with the non-nicking DLE-1 chemistry yielded the most accurate scaffolding, but similar to Hi-C, optical mapping had a limited ability to scaffold short contigs due to the smaller chance of containing sufficient labeled enzyme sequence sites to confidently align the optical cmaps. These small contigs can be difficult to handle in later assembly stages, because most scaffolding tools were not designed to place a contig within an existing scaffold gap. 10XG linked reads were better at localizing smaller contigs, complementing what optical maps missed. We found a few cases where Scaff10X[15] mis-joined contigs due to the ambiguity at the contig boundaries near repeats. However, because subsequent scaffolding and curation steps can break such errors, and because of the difficulty of placing short sequences manually, we kept the linked read scaffolding step before the optical map scaffolding step.

Supplementary Note 2: Species and alternative assemblies

Species were chosen: 1) to compare assemblies of simpler (birds) to more complex and repetitive genomes (amphibians and fishes); 2) to include those threatened (platypus) or critically endangered (kākāpō) of becoming extinct and having low heterozygosity due to small effective population sizes[16]; 3) to answer specific biological questions (e.g. basis of vocal learning in birds and bats)[17,18]; 4) to compare with previous assemblies with available genetic-linkage or FISH karyotype maps (zebra finch, platypus)[6,19]; 5) to contribute to collaborative projects with the VGP (e.g. Bat1K[20]); and 6) to take advantage of high quality samples and available funding within the VGP collaboration. The final assemblies of six species (four teleost fishes, the skate, the caecilian) submitted to the NCBI/ENA public databases (**Supplementary Table 10**) used slightly different pipelines than our standard approach (**Extended Data Fig. 3**). The two species, the thorny skate and channel bull blenny, that did not meet the minimum 1 Mbp NG50 contig size, required manual modifications to the pipeline to do so. Below are brief descriptions of the alternative assembly pipelines used for these species.

The two-lined caecilian, aRhiBiv1.1 (GCA_901001135.1) assembly: This assembly mostly follows the VGP 1.5 standard pipeline, however, run at the Sanger Institute without having a formal functional equivalence evaluation between this setup and the centralized VGP setup. Versions used and differences in the pipeline are as follows: FALCON-Unzip[1] (v1.2.1), Purge_Haplotigs[21] (v1.0.1), Scaff10x[15] (v3.0) (ran two rounds of Scaff10x followed by one round of break10x[15], which differs from the main VGP 1.5 pipeline), Bionano Solve[4] (v3.2.2), SALSA[8] (v2.2), Arrow[11] (GenomicConsensus 2.2.2), longranger[22] (v2.2.2), freebayes[13] (v1.1.0-3-g961e5f3) and bcftools[14] consensus (v1.7). Manual curation was applied as described in the main text and methods and chromosome-scale scaffolds confirmed by the Hi-C data were named in order of size.

The zig-zag eel, fMasArm1.2 (GCA_900634775.2) assembly: FALCON-Unzip[1] (v1.8.6) contigs were created using a read length cutoff of 6,500 bp. The primary contigs were extended by merging with a miniasm[23] (0.2-r168) assembly. The contigs were then scaffolded using the 10XG linked read Illumina data with Scaff10x[15] (v1.0). Further scaffolding was applied using synteny with Lates calcarifer (v3 from http://seabass.sanbi.ac.za/) and the cross_genome tool (https://sourceforge.net/projects/phusion2/files/cross_genome/). PBJelly[9] (PBSuite_15.8.24) was used to fill gaps followed by long read polishing with Arrow[11] (GenomicConsensus 2.2.1). The assembly was polished again using the linked reads by mapping with bwa mem[24] (0.7.17-r1188), calling homozygous non-reference variants with freebayes[13] (v1.1.0-3-g961e5f3) and editing the reference to correct these errors with bcftools[14] consensus (v1.7). This assembly was manually curated with incorporating evidence from Bionano optical map[4] and Arima Hi-C data. This initial fMasArm1.1 (GCA_900634775.1) was submitted as scaffolds. An additional run of SALSA[8] (v2.0) was applied, followed by another round of manual curation to remove heterotypic duplications to produce chromosome-level scaffolds. These chromosome-level scaffolds were named based on synteny to a medaka genome assembly and submitted as fMasArm1.2 (GCA_900634775.2).

The climbing perch, fAnaTes1.2 (GCA_900324465.2) assembly: An initial PacBio contig assembly was made using FALCON-Unzip[1] (v1.8.6) with a read length cutoff of 10,000 bp. The primary contigs were then scaffolded using the 10XG linked read data with two rounds of Scaff10x[15] (v1.0) followed by a round of break10x[15] to break at mis-joins identified by the 10XG data. PBJelly[9] (PBSuite_15.8.24) was used to fill gaps followed by long read polishing with Arrow[11] (GenomicConsensus 2.2.1). The assembly was polished again using the 10XG Illumina data by mapping with bwa mem[24] (0.7.17-r1188), calling homozygous non-reference variants with freebayes[13] (v1.1.0-3-g961e5f3) and editing the reference to correct these errors with bcftools[14] consensus (v1.7). Manual curation incorporated evidence from Bionano optical map and

Arima Hi-C data, and the initial fAnaTes1.1 (GCA_900324465.1) assembly was submitted as scaffolds. An additional run of SALSA[8] (v2.0) was applied, followed by another round of manual curation to remove heterotype duplications using Purge_Haplotigs[21] (v1.0) to produce chromosome-level scaffolds. These chromosome-level scaffolds were named based on synteny to a medaka genome assembly and submitted as fAnaTes1.2 (GCA_900324465.2).

The channel bull blenny, fCotGob3.1 (GCA_900634415.1) assembly: An initial PacBio Falcon-unzip[1] (falcon-2018.03.12-04.00) assembly was run without Dazzler repeat-masking during overlap detection. A separate wtdbg[25] (v1.1) assembly was made from the PacBio reads. Contigs from the wtdbg assembly were used to guide initial scaffolding of the Falcon contigs using cross_genome, then scaffolded further with the 10XG Illumina data and Scaff10x[15] (v1.0). The Bionano optical map data was used for two-enzyme hybrid scaffolding[4] (Solve3.2.2_08222018). The PacBio CLR data was used to gap fill with PBJelly[9] (PBSuite_15.8.24) and polish with Arrow[11] (GenomicConsensus 2.2.2). The assembly was polished again using the 10XG Illumina data by mapping with bwa mem[24] (0.7.17-r1188), calling homozygous non-reference variants with freebayes[13] (v1.1.0-3-g961e5f3) and editing the reference to correct these errors with bcftools[14] consensus (v1.7). The assembly failed to meet the VGP contig NG50 goals, so a new strategy was tried to improve the assembly. Canu[26] v1.6 was used to correct the PacBio reads using a k-mer size of k=21. Contigs from a wtdbg[25] (v1.1) assembly of the corrected reads were then used to conservatively fill gaps in the main assembly where contigs were unambiguously anchored on either side of a gap. Long-read and short-read polishing was performed as above, to ensure sequence that had been used to fill gaps was also polished. Retained haplotigs were identified and removed with Purge_Haplotigs[21] (v1.0). Finally, the assembly was scaffolded to chromosomes using Arima Hi-C data and SALSA[8] (v2.0). Manual curation was applied using gEVAL[27] as described in the main text and methods to correct mis-joins and improve concordance with the Bionano optical map and Arima Hi-C data. This assembly met the VGP metrics, and chromosome-scale scaffolds were named based on synteny to a medaka genome assembly.

The eastern happy, fAstCal1.2 (GCA_900246225.3) assembly: First the PacBio raw reads were scrubbed to remove chimeric reads and other artifacts using the Dazzler framework (https://dazzlerblog.wordpress.com/2017/04/22/1344/). The scrubbed reads were then used to make an initial contig assembly with PacBio Falcon-unzip (https://github.com/millanek/FALCON-integrate). A separate assembly was created with miniasm[23] (0.2-r159), then used to scaffold the Falcon primary contigs using cross_genome. The contigs were then scaffolded further using the 10XG Illumina data with Scaff10x[15] (v1.0). Some contigs in the scaffolds were gap filled with PBJelly[9] (PBSuite_15.8.24) and polished with Quiver[11] (GenomicConsensus 2.2.1). The assembly was manually curated using gEVAL[27] to correct mis-joins and improve concordance with the Bionano optical map data. The assembly was then polished again using the 10XG Illumina data by mapping with bwa mem[24] (0.7.17-r1188), calling homozygous non-reference variants with freebayes[13] (v1.1.0-3-g961e5f3) and editing the reference to correct these errors with bcftools[14] consensus (v1.7). This assembly was submitted as fAstCal1.1 (GCA_900246225.1). A further round of curation and verification with gEVAL[27] along with integration with two genetic maps[28,29] allowed assignment of scaffolds to chromosomal linkage groups. This was submitted as revised assembly fAstCal1.2 (GCA_900246225.3).

The thorny skate, sAmbRad1.pri (GCA_010909765.1) assembly: Applying the VGP 1.0 pipeline to the thorny skate did not result in an assembly that met all the desired VGP metrics, due to the very high repeat content in this species, as described in the main text. Therefore, we developed a modified approach that handled high repeat genomes better. We used Canu[26] v1.7 to assemble the contigs, and purged false duplications with Purge_Haplotigs[21] instead of the purged FALCON-Unzip[1] contigs, because the contig NG50 and overall BUSCO[30] completeness scores were higher in the Canu contigs (**Supplementary Table 13**, compare p1 stats of the vgp_standard_1.5 and vgp_nhgri_1.5). We believe these differences could be due to the less aggressive repeat masking of Canu compared to FALCON. The rest of the scaffolding process

followed the VGP Standard Pipeline 1.5. Two rounds of Arrow[11] polishing was applied (t1), with 3 rounds of SR polishing (t2~t4) with the 10XG linked reads using longranger[22] align (v2.2.2) and freebayes[13] (v1.3.1) `--skip-coverage` option to skip regions with excessive coverage. Too many false duplications were found during curation, and thus the assembly was sent back for further improvements. We applied Purge_Dups[31] on t4 primary scaffold, by breaking scaffolds at any gaps. Purged primary contigs (u1) were re-scaffolded with optical maps (u2) and further scaffolded with 2 rounds of SALSA[8] (u3-u4). We discovered a linked read library failure and so obtained additional Illumina WGS reads. Using the WGS reads, 3 rounds of polishing was performed with bwa mem[24] and freebayes.

Supplementary Note 3: Resolving missing genomic regions

Although the VGP genomes have a greater amount of genomic content assembled relative to the most commonly used prior references (e.g. **Extended Data Fig. 8**), we also noted that some of the VGP genomes had a smaller proportion of missing genomic content relative to prior assemblies. We investigated this source of the missing regions, and found it was due to repeat content and GC-content. Almost all genome assemblers mask out reads with repeat structure before assembly, as it becomes computationally expensive or impossible to assemble with repeats present. FALCON masks portions of reads which coincide with repetitive regions, and does so in two stages: 1) tandem repeat masking; and 2) masking of general repeats/segmental duplications. The masked repetitive regions then do not contribute to the overall overlap computation. For large repeats (longer than the read length), this means that the genomic region will not be represented in the final assembled contigs. In addition, and not related to repeat masking, FALCON also applies a cutoff threshold to limit the minimum length of reads to find overlaps. If the limit is set too high, the assembly may miss some genomic regions. After the initial contig phase, the repeats as well as smaller reads are brought back into the assembly for Arrow[11] polishing and later gap filling. However, we found that certain reads with repeats and a given read size were not being incorporated into the assembly if they did not have a region to anchor onto in the initial contigs. An example were some genes with GC-rich sequence and repeat regions of the Anna's hummingbird that were present on reads shorter than 10,000 bp. These non-repetitive genes were surrounded by GC-rich and repeat genomic regions, which may have biased the molecule size of the sequencing library[32]. These shorter and/or repetitive reads were excluded from overlap detection, or ignored due to the shorter overlap length with no anchor to bring them into the assembly at later stages. When we reduced the p-read cut off to 2,000 bp, the NG50 values decreased, but many of the genes on these shorter and repetitive reads were incorporated into the assembly. This highlights the need of further investigation and improvements in ways to rescue missing regions when applying general length cutoffs during the assembly process.

Supplementary Note 4: Assessing overall assembly quality

Below are example measures for the six categories of genome assembly quality proposed in this study (**Table 1**).

Continuity: The current most popular measure of genome assembly continuity is the ***scaffold N50***, and secondarily the ***contig N50***, defined as the largest *s* where scaffolds (or contigs) of length *s* or greater is half or greater the total assembly size. However, the assembly size can be larger or smaller than the true genome size depending on the assembly tools and data quality used. Thus, we recommend using *NG50* (G

for genome), which uses the estimated genome size instead of the assembly size for normalization[33]. We prefer to estimate the genome size from actual sequence data, using *k-mers* (sequence fragment length of *k*) such as done in GenomeScope[34]. All high copy *k-mers* should be included when counting *k-mers* to properly include repeat contents, which is not a default behavior in most *k-mer* counters for practical reasons. For gaps, we recommend using a measure of the rate of ***gaps per unit of Gbp*** assembled, as larger genomes would otherwise be penalized for containing more gaps.

The specific metric thresholds we chose for the continuity measures were based on output of the achievable short- and long-read based assembly pipelines we assessed. In the B10K-2014 short-read only assemblies, protein coding exon sequences are mostly complete, but they often have incomplete exon-intron gene structure, missing GC-rich regulatory regions, and/or genes with high repeat content. In the VGP-2016 to VGP-2020 assemblies, most gene structures have high continuity, but the highly repetitive centromeres and telomeres may be incomplete. The thresholds of the finished quality assemblies were calculated based on gapless and error-free assembled chromosomes, with complete, non-collapsed centromeres, telomeres, and other segmental duplications.

Structural accuracy: To assess structural accuracy without a known truth, we propose mapping the raw data types to the final assembly and measure concordance as the NG50 size of ***reliable blocks***. We define concordance here as how many of the data types support the assembled structure at each base. In this study, we defined reliable blocks with support from at least two of the four sequencing platforms (long reads, linked reads, Opt, and Hi-C). This can be extended with additional data types, such as genetic maps or FISH karyotypes, when available. In assemblies that only have one or two data types, it is more difficult to determine reliability, but one can still consider consensus read data from high-coverage sequencing as another measure of both sequence and structural reliability.

Each supportive region is obtained by mapping back sequencing data to the assembly. Regions with excessive coverage or coverage dropouts are usually an indication of an assembly error and thus get excluded. Regions with excessive coverage are caused by collapsed bases, where sequences are present in the assembly with an unexpected copy number. Coverage dropouts are caused by chimeric junctions. To measure these structural metrics, we used an implementation in Asset[35], with details described in the **Supplementary Methods**.

Our third measure of structural accuracy is ***false duplications***. Potential false duplications are often reflected in the BUSCO duplication score. But to assess false duplications with species that are highly divergent from the available BUSCO database, we also use *k-mers*. For a *k-mer* size that is sufficiently long to be unique in the genome, and a genome sequenced with high-fidelity reads to a depth of coverage *c*, a complete *de novo* assembly should recover *k-mers* from the homozygous (two-copy) regions of the genome with roughly *c* times and *k-mers* from the heterozygous (single-copy) regions with *c/2* times. All *k-mers* in the heterozygous and homozygous regions are expected to be found once in a (pseudo) haplotype assembly. Any additional *k-mer* copy found in the assembly compared with the high-fidelity reads are considered to be falsely duplicated[36]. To identify falsely duplicated *k-mers*, we used an implementation in Merqury[37] to count the number of distinct *k-mers* with additional copies in the heterozygous and homozygous regions and report the relative portion compared to the expected *k-mers* with no additional copies (**Supplementary Fig. 2**). Both approaches (BUSCO and *k-mer*) showed comparable trends, with the *k-mer* approach having higher sensitivity as it captures false duplications on a genome-wide level (**Fig. 2f-i**).

Finally, ***curation*** of the genome assembly manually assesses structural accuracy of the automatically-generated assembly, identifying false chimeric joins (misjoins), missed joins, inversions, and other errors. When fixed by a manual or automated process, this increases the structural accuracy of an assembly.

The specific thresholds we chose in each quality category reflects the range of NG50 reliable blocks we obtained with the 16 species in this study and the false duplication rate, which is influenced by the degree of correct haplotype phasing. For the VGP quality assemblies, we listed a manual curation

process, which is valuable and essential as highlighted in this study. We caution that it is subject to individual human interpretation and requires specialized expertise. To scale up to 1000s of genomes, the curation process would benefit from more automated processes to identify and fix structural errors, which is currently an active area of development. More details on each curation step and tested improvements are described in our companion paper by Howe et al[38].

Base accuracy: There are multiple ways of measuring base-level accuracy, called ***base pair QV***. One approach is to align (i.e map) highly accurate reads to the assembled genome and call base errors similarly to variant calling. We define "mappable" as all reads that align, excluding low-coverage and excessively high-coverage regions (see **Supplementary Methods** for exact parameters used), where we can rely on base error calls. The other, more reliable, way to measure base accuracy is using *k-mers* found both in the assembly and highly accurate unassembled reads. Base error rate inferred directly from *k-mers* was more comprehensive, and thus more accurate than the widely used mapping and variant calling protocols, which artificially inflated QV values because they excluded regions that are difficult to map (**Supplementary Table 17**). All *k-mers* found only in an assembly are likely produced from a base pair error. By counting these *k-mers* and comparing the fraction to all *k-mers* found in an assembly, we can estimate the error rate and calculate the quality value using the *k-mer* survival rate[37]. We found *k-mer* based methods include unmappable regions and thus avoid over-estimated QVs from the mapping-based approach. We note that both mapping-based and *k-mer*-based approaches have limitations of measuring base accuracy in highly repetitive regions, as the short reads are difficult to map accurately and a *k-mer* with a true error may match by chance with some other true *k-mer* that belongs elsewhere in the genome. This may artificially inflate the QV, especially in those repetitive regions.

To assess if all bases in a genome are properly assembled, we propose using *k-mers* as the truth set to get an estimate of ***k-mer completeness***. Reliable *k-mers* obtained from highly accurate reads are obtained by excluding erroneous *k-mers* from sequencing errors. The fraction of the *k-mers* found in the assembly of these reliable *k-mers* are indicative for genome completeness. This measure is dependent on the base pair QV as well, because *k-mers* from assembly errors will affect the completeness measure. We use the implementation in Merqury[37] to obtain the *k-mer* completeness, reported in **Extended Data Table 1**.

The specific thresholds we chose reflects the level of tolerance one is willing to have for nucleotide errors, which can be misinterpreted as biological variation. A Q40 value means an average frequency of 1 error every 10,000 bp, which means that genes this size or bigger are likely to have at least 1 error. A Q50 value, 1 error in every 100,000 bp, which is equivalent to a large multi-exon gene, means that most genes will be unlikely to contain an error within their coding sequence. The *k-mer* completeness thresholds are a reflection of the *k-mer* based QV thresholds, and give a sense of the base level accuracy of the genomes assembly as a whole.

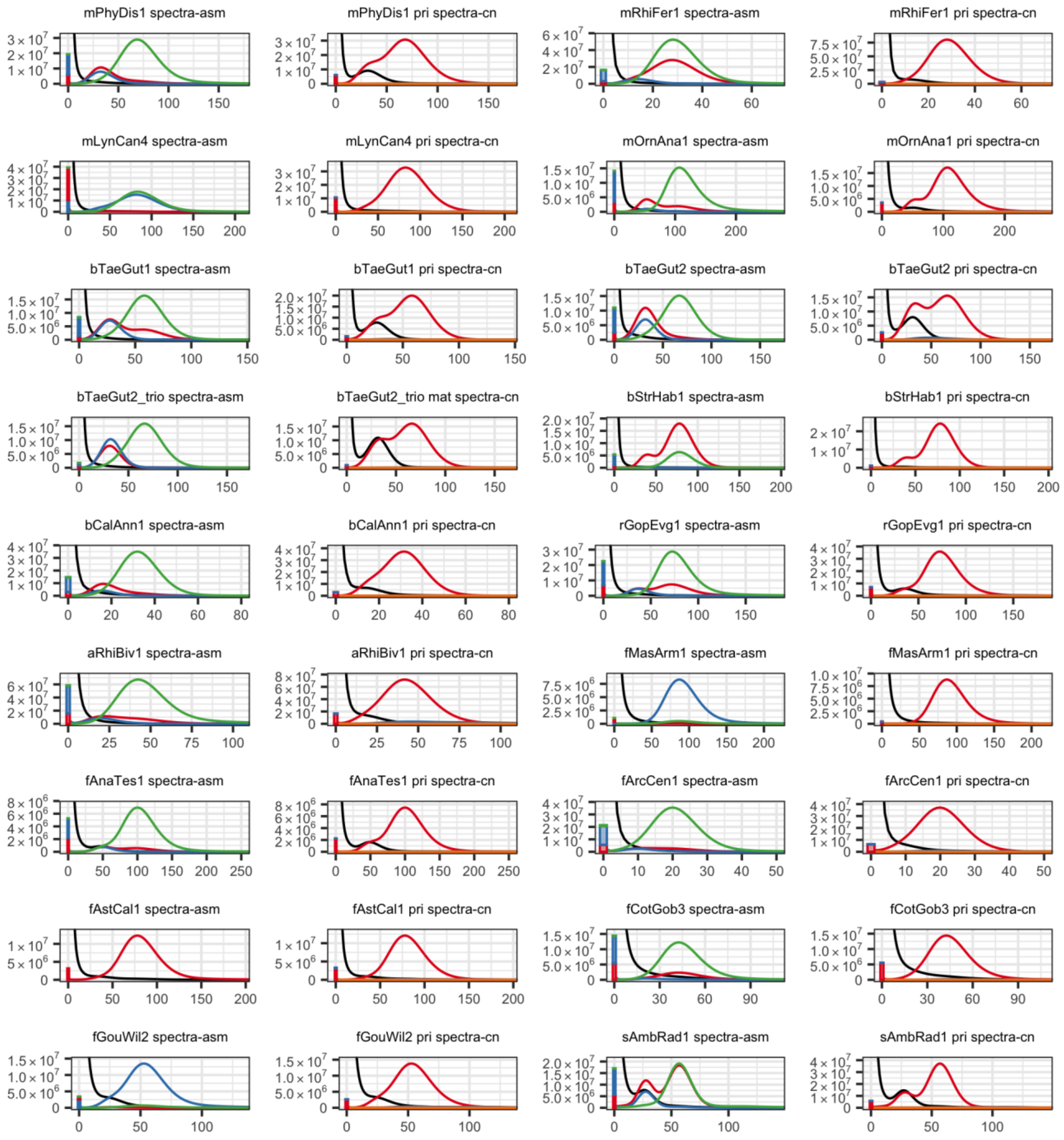

Supplementary Fig. 2 | *k-mer* spectrum of each submitted assembly, plotted on the 21-mer multiplicity found in the Illumina sequencing set from the 10X linked reads. For each species, the first and third column of graphs show the overall *k-mers* found (spectra-asm) in the primary set (red), alternate set (blue), shared in both assemblies (green), and missing *k-mers* in any assembly set (black). Fewer missing *k-mers* observed (black) indicates the assembly more completely represents the genome. The second and fourth columns show the copy number spectrum (spectra-cn) of the primary assembly set, colored by the copy numbers found in the assembly: once (red), twice (blue), 3 (green), 4 (purple), >4 (orange), and missing (black). *k-mers* in spectra-cn are expected to be found once (red) in a pseudo haplotype assembly; thus, *k-mers* found more than once (blue, green, purple, and orange) originate from falsely duplicated sequences assuming no allele-specific duplications exist in the genome.

Haplotype phasing: We propose to use ***phase block NG50*** as a measure for haplotype consistency. A phase block is expected to match one of the parental haplotype sequences, with no haplotype switches. Haplotype consistency is important for gene annotation, because haplotype switches could mix the true gene structure, creating an artificially mixed gene that does not exist in nature. Currently, the most reliable way to measure phase consistency is by using parental sequences. In this study, we used Merqury[37] to infer haplotype blocks from haplotype specific *k-mers*. Accounting for sequencing errors accidentally corrupting a true haplotype specific *k-mer*, we allowed short-range switches to occur up to 0.05% (~100 times within 20kbp) within a phase block. We expect block sizes to be more dependent on genome heterozygosity levels, where less heterozygous genomes will have longer runs of homozygosity (ROH) that prevent linking of heterozygous sites when no parental information is used. Heterozygosity will also vary across segments of a genome, and thus, one value may not be equally applicable across the genome. Therefore, we set smaller block NG50 requirements to cover one gene and its regulatory regions (typically 10 to 500 kbp) in one phase block, which falls within 1Mbp NG50 in the quality metric (**Table 1**), independent of chromosome sizes except for the "finished" quality.

Methods for cross linking distant heterozygous sites using Hi-C or Strand-seq are on the horizon[2,39,40], which will help increase phase block continuity. However, accurate measures of the phase blocks are not as well developed without parental data, presumably because the importance of phasing to prevent errors has been unappreciated. This measure pertains to not only diploid genomes, but also polyploid genomes, which are found in amphibians and fishes.

Functional completeness: ***Gene-based metrics*** could be used as an indicator for genome completeness and is one of the most important factors when conducting functional studies. However, it is almost impossible to have a truth set of all genes, especially for genomes with no reference available. One indirect way to measure functional completeness is by using BUSCO genes sets, which are sets of highly conserved orthologous genes present in a single copy across vertebrates or other groups of species[30]. To work properly, the sequences of the gene set needs to be complete and error free, but this is not the case for many BUSCO genes[41]. Overall, however, the absence of complete single copy genes in an assembly may be evidence of functional incompleteness.

Transcript mappability with transcriptome data from the same species or even individual is a more robust way to measure gene completeness, because a more complete genome is expected to map more transcriptome sequences unambiguously (uniquely) to the assembly. In addition to transcripts, one can assess functional regulatory genome completeness by mapping epigenetic sequence data, as we have done here with ATAC-Seq reads (**Fig. 3a-b and 4c-d**).

Considering the thresholds we chose, without BUSCO gene sets being complete, and with natural gene losses in some species, there will be an upper limit of less than 100% mapping for some species, and this is why we chose 98% in the finished quality category. For our VGP assemblies, we have obtained some assemblies with 99.9% BUSCO gene mapping. The thresholds for transcriptome mappability were determined based on empirical observations shown here, and align with the BUSCO scores in some assemblies. The epigenetic genome mapping scores for the zebra finch assemblies were lower than for the transcripts, and this we believe could be due to regulatory regions having a higher GC content, which can be harder to sequence and assemble.

Chromosome status: For defining scaffolds as chromosomes, and therefore the ***percent of the assembly assigned to chromosomes***, we believe the current best tool besides genetic linkage or FISH karyotype mapping is Hi-C mapping. We consider a scaffold as a complete chromosome (albeit with gaps) when there is a diagonal signal in the Hi-C mapping plot for that scaffold with no other scaffolds that can be placed in that same scaffold. The Hi-C maps prove useful for identifying large-scale structural aberrations in the assemblies, including false chromosome fusions. The more uniform the Hi-C signal across the main diagonal, the more likely the assembly structure is correct. High-frequency, off-diagonal Hi-C interactions are a strong sign of mis-assembly, of which some can be corrected with manual curation. Based on these

criteria, one can then estimate the percent of the genome that is assigned to chromosomes. The thresholds we set from 75% to 100% chromosome assigned are based on values we generated in this study using different assembly approaches. See Lewin et al.[42] for an alternate view of naming scaffolds.

Sex chromosomes are typically a challenge as they are often highly diverged between the partners. The sex-specific chromosome (e.g. Y in XY mammals or W in ZW birds and snakes) are often rich in highly repetitive heterochromatin. Sex determination mechanisms are highly variable in amphibians, reptiles, and fishes, with different sex genes (mostly unknown) defining non-homologous sex pairs. In many species, it is unclear whether there is male heterogamety (XY as in mammal) or female heterogamety (ZW as in birds). Many reptiles and some fish have no sex chromosomes and determine sex by an environmental signal (commonly temperature). Thus, we only require sex chromosomes to be assembled and identified in lineages known to have sex chromosomes, and make an effort to sequence the heterogametic sex to assemble both sex chromosomes, or one of each sex to have greater confidence. Once a pseudo-haplotype assembly is assembled, sex-specific chromosomes can be further determined by comparing differences in read depth in males and females when available, identification of known of sex-specific genes for the relevant clade, synteny with sex chromosomes in closely related species, and the coverage pattern of PAR and haploid regions.

Organelle genomes, as shown here, can require a different set of tools to assemble, but are still subject to some of the same metrics. This includes QV, scaffold, contig, gaps, and other values. As shown here, obtaining one complete gapless and accurate assembled sequence is possible with mitochondrial genomes using a combination of short and long reads, due to the relatively smaller size of its genome.

Supplementary Note 5: The Vertebrate Genomes Project

The goal of the Vertebrate Genomes Project (VGP) is to generate at least one high-quality, error-free, near gapless, chromosome-level, haplotype phased, and annotated reference genome assembly for all extant vertebrate species and to use those genomes to address fundamental questions in evolution, disease, and biodiversity conservation. We plan to conduct this international project in phases according to phylogenetic scale, from orders (Phase 1) to families (Phase 2), genera (Phase 3), and finally all species (Phase 4; **Supplementary Fig. 3**). Phase 1 serves as a proof of principle project. At the family level, we would complete vertebrates in the Phase 1 goal of the Earth BioGenome Project (EBP BioProject ID PRJNA533106) for high-quality reference genome assemblies for all eukaryotic families[43]. At the genus level, we would complete the original G10K mission of approximately 10,000 vertebrate species[44]. At the final species level, we would complete the data generation mission of the VGP (BioProject ID PRJNA489243) and specific vertebrate taxonomic groups, such as all birds (B10K[6,45] BioProject PRJNA489244) and all bats (Bat1K[20,46] BioProject PRJNA489245).

For Phase 1, although there are approximately 150 named orders of vertebrates, the criteria for taxonomic divisions are not consistently applied among vertebrate classes. Therefore, we sought to use a more uniform definition. Based on findings from the Avian Phylogenomics Project[47] and mammalian phylogenomic studies[48], we noted that taxonomists have often delimited orders encompassing species that shared a most recent common ancestor 50-70 million years ago (Mya), following the last mass extinction event at the Cretaceous-Paleogene transition. Thus, for VGP Phase 1 we aimed to partition lineages that have an inferred common ancestor not substantially older than 50 Mya. This definition resulted in our current target list of approximately 260 "order level" lineages (http://vgpdb.snu.ac.kr/details/).

When we first began working on the hummingbird assemblies in 2015 and initiated the VGP and sequencing of ordinal representative genomes in 2017, there were 66,178 named species gathered from various databases, estimated based on the IUCN Red List of Threatened Species and reported in the Vertebrate Wikipedia page from 2014 to date (https://en.wikipedia.org/wiki/Vertebrate). This is a number that we had initially used in public announcements of the project[49]. However, we collated available lists of vertebrate species, and we obtained 71,657 named species as of January 2019. We believe the increased number of species is due to additional species discoveries in the last 10 years, revisions of previously defined species (e.g. Northern vs. Southern ostrich), and analyses of genomic relationships[50]. With this list, we have created, for the first time that we are aware of, an all-vertebrate species list (http://vgpdb.snu.ac.kr/splist/). We are populating this list with accessions to the high-quality reference genomes, including the 17 of this study, as well as draft and medium quality genome assemblies. We hope that this list will be useful to the scientific community to track genome assemblies for all vertebrate species.

To conduct the VGP in an efficient and democratic manner, we built a governance and committee structure that consists of an executive council and task-specific committees focused on specific issues, including permits, sample preparation, genome assembly, genome annotation, comparative genomics, and conservation genomics. We developed a scalable assembly pipeline within a cloud environment, where working data is hosted on an Amazon S3 bucket (s3://genomeark). The production of the VGP assemblies is performed on DNAnexus, which is available for anyone. The entire source code of the pipeline to run locally or on the DNAnexus platform is publicly available on github (https://github.com/VGP/vgp-assembly). The scaffolding pipeline is also available to run on a generic compute architecture using Docker containers (**Supplementary Note 7**). Intermediate assemblies and raw data are available to download from Genome Ark (https://vgp.github.io) until archived in an INSDC database (e.g. GenBank). We also built a public website for the VGP (https://vertebrategenomesproject.org/), and its parent G10K (https://genome10k.soe.ucsc.edu/), with links to associated projects (B10K, Bat1K, and EBP). Our assemblies and raw sequences are deposited in international public databases with NCBI and EBI under a VGP BioProject ID PRJNA489243

(https://www.ncbi.nlm.nih.gov/bioproject/489243). We currently produce about three genome assemblies per week, but will need to scale up to 125 genomes per week to complete all ~70,000 species within a 10-year period, assuming the availability of future funding and the development of more advanced computational infrastructure. In addition to the 17 genomes released publicly with this publication, there are 120 more assemblies in progress (https://docs.google.com/spreadsheets/d/1s5J-s3Tat3U_wQcik_xhVHwH6AAXr5D9AMRu-e22XDw/edit?usp=sharing), that are supported by individual institutions and scientists (https://genome10k.soe.ucsc.edu/data-use-policies/).

The challenges for scaling up that we are working on include: 1) Blanket sample permits for vertebrates, in different countries; 2) High throughput DNA and library sample preparations for ultra-high molecular weight DNA (>200kb); 3) An automated metadata tracking system for information flow from samples to their genomic data, transcriptomic data, assemblies, and annotations; 4) Increased efficiency to perform massively parallel high-quality sequencing; 5) Automated assembly pipeline that allows iterative updates, and more efficient assembly compute for hundreds of assemblies simultaneously; 6) A more automated curation process and many curators to manually check each assembly, make fixes where needed, and provide iterative feedback; 7) A more efficient reference-free genome alignment tool that can handle 10,000s of species; and 8) Rapid annotations of genomes in the hundreds per week. The VGP is working on all eight fronts, with the plan that at each Phase of the project will need more and more advanced tools for increased scaling. Future efforts should also include development of tools that can automatically estimate parameters needed to assemble a genome accurately with different repeat, heterozygosity, and ploidy levels.

Related large-scale reference genome efforts have adopted lessons learned from the VGP, including the Bat1K[20,46] (https://bat1k.com), Bird B10K[6,45] (https://b10k.genomics.cn), Global Ant Genomics Alliance[51] (GAGA; http://antgenomics.dk), Earth BioGenome Project[43] (EBP. https://www.earthbiogenome.org), Global Invertebrate Genomics Alliance[52] (GIGA; http://giga-cos.org), Darwin Tree of Life (https://www.darwintreeoflife.org), and human pangenome (https://humanpangenome.org) projects, which target all species for particular clades or geographic regions of interest, or multiple individuals within a species representing diversity of the extant population.

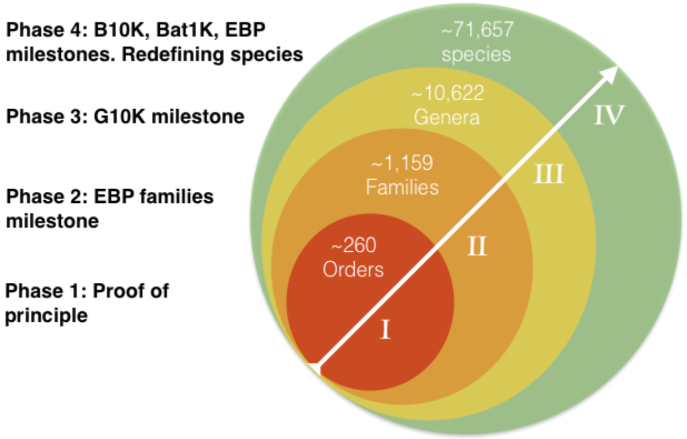

Supplementary Fig. 3 | Schematic of proposed phases to conduct the VGP. Circles represent phylogenetic classification scales, going from smaller to larger numbers of species (arrow). A sequenced species represents an order, family, and genera in Phases 1-3. To the left are listed goals and related projects for whose milestones will be completed at the completion of specific VGP phases. Redefining species means that within Phase 4 it might become possible to use the genome sequence differences to determine when individuals should be considered belonging to the same species or their own distinct species[50].

Supplementary Note 6: Current advances in technologies

After we completed the evaluations on the genomes of the 16 species in this study, as expected, new advances in genome sequencing technology and algorithm development have been made. Here we provide a prospective on these developments, including how we will potentially incorporate them into a Phase 2 VGP pipeline, towards even higher-quality assemblies.

The 10XG linked read technology we used is no longer available as of mid-2020, but can be substituted by three other technologies: TELL-Seq WGS (Universal Sequencing)[53]; stLFR (BGI)[54]; and CPTv2-seq (Illumina)[55]. The ONT data generated here were not considered for further benchmarking beyond contigs due to practical issues concerning systematic base call errors, consistency, and scalability at the time (early 2017)[56]. However, the technology has since improved in these areas[57], and the latest r10.3 base calling leads to higher-quality long reads. These could be potentially in place of or in combination with the PacBio long reads. PacBio has also recently introduced their next generation reads, circular consensus reads (CCS) or HiFi[58], which delivers both reasonable read length (20 kbp) and excellent read accuracy (99.9%) in a single technology. The higher accuracy may eliminate the need for assembly polishing. The VGP infrastructure has been flexibly designed so that we may continually evaluate and adapt to new technologies in order to reach our ultimate goal of producing error-free, gapless, and complete telomere-to-telomere assemblies. We believe the principles of the pipeline we generated will be applicable to new technologies. We are optimistic that, given continuing advances in diploid sequencing and assembling technology, finished-quality reference genomes will be achievable at reasonable cost for most species of interest within the next decade.

Supplementary Note 7: Assembly pipeline using Docker

An implementation of the pipeline has been developed to run on generic architecture using WDL workflows (https://github.com/openwdl/wdl/blob/master/versions/1.0/SPEC.md) and Docker containers (https://www.docker.com/). We intend for this to be a portable and modular implementation, which diverges from the main workflow as little as possible.

WDL (Workflow Description Language) is a standard which enables the description of workflows in both human- and machine-readable ways. Workflows are composed of tasks; tasks have defined inputs and outputs, a script to perform the work, hardware requirements, and an environment in which to be run. Docker is a virtualization tool that we use to provide the environment for tasks. A Docker container is a lightweight image of a filesystem that can contain specific tool versions. It uses a layered filesystem, where multiple snapshots can inherit from a single base image.

The design of the VGP's WDL workflow implementation aims to replicate current functionality while minimizing changes to the main codebase. The main codebase is designed to run in an HPC environment and uses CEA-HPC Modules (https://github.com/cea-hpc/modules) to manage use of specific tool versions. The WDL implementation replicates this environment in the Docker images, so as to reduce modification to the existing scripts. There is a base Docker image which includes the modules infrastructure, common libraries, and tools used in multiple tasks. Task-level Docker images extend from this and add task-specific code. Slurm submission scripts from the original pipeline were rewritten and translated into WDL tasks. Operative bash scripts (the entry points Slurm uses) are copied into the task images and are invoked directly where possible.

Scaffolding and QC tasks have been implemented in WDL/Docker: Contigging+purging, linked read scaffolding, optical map scaffolding, Hi-C scaffolding, BUSCO[30], and Merqury[37]. Each task can be run independently, and the whole scaffolding suite can be run via a single workflow. For information on running the workflow, see the manual here:

https://github.com/VGP/vgp-assembly/blob/master/wdl_pipeline/WDL_Manual.md

Supplementary Methods

Quality control and contamination screening

Before assembly of the sequence and scaffold data, a quality control screening for poor sequencing reactions or contamination with foreign genome data was performed using Mash. When running Mash[59], 21-mers were used to generate sketches with sketch size of 10,000 and compared among each sequencing runs. For example, using this approach, we detected two outlier libraries in the initial Canada lynx PacBio data that did not cluster with the other sequencing runs (**Supplementary Figure 4**). Further investigation determined that these files had been mis-tracked and the data originated from an unrelated sequencing project on rice, so the rice runs were removed prior to assembly.

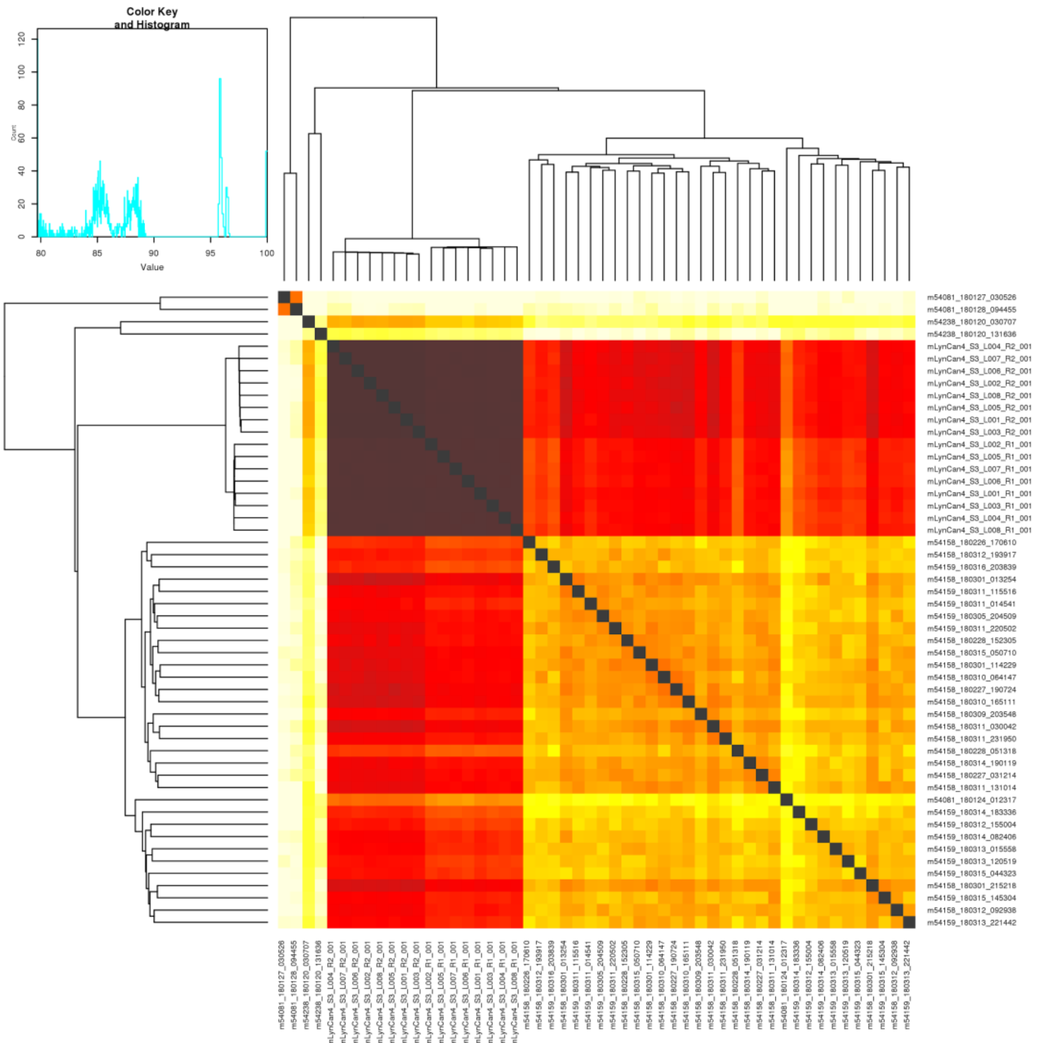

Supplementary Fig. 4 | Mash maps to detect poor quality data and foreign species contamination. Raw read data of PacBio CLR (top left 4 and the bottom right) and linked reads (darker brown area) are aligned to each other. Based on sequence similarity and coverage, the top two (top left corner) SMRT cells were identified as outliers. The higher the similarity and coverage, the darker the color.

Binning 10XG linked reads and Hi-C reads

For the trio based assembly, linked reads and Hi-C reads were binned using an "exclusion" criteria. Unlike trio-binning[3], here we excluded any read-pair having at least one parental specific k-mer when assigning to a bin. This allowed short reads to remain in both maternal and paternal bins, which originate from the homozygous part of the genome. This process uses `meryl-lookup` in Meryl[37] v1.0:

```
meryl-lookup -memory 2 -exclude -mers pat.meryl -sequence $read1 -sequence2 $read2 -r2
mat.R2.fastq.gz | pigz -c > mat.R1.fastq.gz
meryl-lookup -memory 2 -exclude -mers mat.meryl -sequence $read1 -sequence2 $read2 -r2
pat.R2.fastq.gz | pigz -c > pat.R1.fastq.gz
```

An implemented version is available on Merqury:

https://github.com/marbl/merqury/blob/master/trio/exclude_reads.sh

ENSEMBL annotation pipeline

The Ensembl gene annotation system[60] was used to generate annotation for the high-quality assemblies. Annotation was created primarily through alignment of transcriptomic data to the genome, with gap filling via protein-to-genome alignments of a select set of vertebrate proteins from UniProt[61] and, for mammal species, coordinate mapping of GENCODE[62] human reference annotations via a pairwise whole genome alignment.

The transcriptomic data consisted primarily of short-read RNASeq data sourced from the public archives, which included data for most species generated by the VGP for this study. This included PacBio Iso-Seq and Nanopore long-read transcriptome data. Short-read data were initially mapped via bwa mem[24] and then locally re-aligned in a splice-aware manner via Exonerate[63]. Transcripts were then inferred based on the strongest intron/exon signals for likely genic loci on a per tissue basis. Long-read data were mapped to the genome using Minimap2[64] with the recommended settings for Iso-Seq and Nanopore data. Due to the high error rate of the Nanopore data, post mapping error correction was employed to maximize the number of usable mappings. Intron/exon boundaries that were non-canonical or deemed low frequency (five or fewer observations across all mappings at a locus) were replaced with high frequency boundary coordinates (greater than five observations) within a 50bp edit distance. High frequency boundary observations were determined both from canonical boundary observations from the Nanopore mapping themselves and also from the alignments of the short-read data. A similar strategy was employed to remove likely artificial gaps of 200bp or less from exons described by the Nanopore data. In these cases, low frequency potential gaps between two adjoining exons were filled in based on high frequency observations of single exons with the same terminal boundary coordinates. For each transcript model generated from either short- or long-read data, the longest open reading frame was assessed via a BLAST[65] of UniProt vertebrate proteins that had experimental evidence of existence at either the protein level or the transcript level.

For gap filling, where the transcriptomic data were absent or fragmented, homology-based methods were employed. Splice-aware protein-to-genome alignments were carried out via GenBlastG[66]. Annotation mapping from human was carried out via a pairwise alignment using LastZ[67] (https://etda.libraries.psu.edu/catalog/7971) and subsequent exon coordinate mapping and transcript reconstruction via both in-house software and CESAR[68] v2.0.

At each locus, low quality transcript models (in particular those with evidence of a fragmented ORF) were removed, and the data collapsed and consolidated into a final gene model plus its associated non-redundant transcript set. ORF likelihood was determined by aligning the ORF translation against known vertebrate proteins. Priority was given to models derived from transcriptomic data. For loci where

the transcriptomic data was not available or highly fragmented, homology data took precedence, with preference given to longer transcripts that had strong intron support from the short-read data. Summary statistics of the annotations are available in the annotation reports in **Supplementary Table 20**.

NCBI annotation pipeline

The NCBI Eukaryotic Genome Annotation Pipeline was used to annotate genes, transcripts, and proteins on the primary assembly of the 17 assemblies, submitted between September 2018 and April 2020, and the product of the annotations were added to the RefSeq collection. The genome sequences were masked using Windowmasker[69]. RNA-Seq reads were retrieved from SRA for the species, or for the family, in the case of lynx, kakapo, Anna's hummingbird, Goode's thornscrub, blunt snouted clingfish and thorny skate for which no or insufficient amount of RNA-Seq data was not yet available at the species level. Depending on the species, between 385 million and 10 billion RNA-Seq reads, ESTs, and RefSeq and GenBank transcripts for the species or closely related species were aligned to the masked genome using BLAST[65] followed by Splign[70]. PacBio IsoSeq for lynx, kakapo, Anna's hummingbird, zebra finch and Oxford Nanopore Technologies transcriptomics reads for kakapo were aligned to these species' respective assemblies using minimap2[64]. In addition, human RefSeq proteins and GenBank and RefSeq proteins for related organisms were aligned to the genome using Blast and ProSplign. The gene models' structures and boundaries were obtained with Gnomon(NCBI eukaryotic gene prediction tool. Available at: https://www.ncbi.nlm.nih.gov/genome/annotation_euk/gnomon/) by "chaining" the alignments into preliminary models. Partial open reading frames on these chained alignments (missing either a start or a stop) were joined and filled if adjacent and in compatible frames, or extended by the ab initio module of Gnomon using a hidden Markov model trained on the species, if the coding propensity of the region was sufficiently high, or called non-coding. tRNAs were predicted with tRNAscan-SE:1.23[71] and small non-coding RNAs were predicted by searching the RFAM 12.0 HMMs for eukaryotes using cmsearch from the Infernal package[72].

Gene and transcript models were then evaluated at each locus, and one of overlapping gene models was chosen in this order of precedence: same-species curated RefSeq models, RFAM models, Gnomon models, and finally tRNA models. Among Gnomon models, if multiple fully-supported transcript variants were predicted for a gene, only the models supported in their entirety by a single long alignment (e.g., a full-length mRNA) or by RNA-Seq reads from a single BioSample were selected. Poorly supported Gnomon models conflicting with better-supported models annotated on the opposite strand were excluded from the final set of models. Further filtering of gene models, and assignment of function, name and type to the final accepted gene set was based on orthology to human genes (or zebrafish in the case of the climbing perch) and Blast hits to SwissProt or, as a last resort, Blast hits to nr. Gnomon models with high homology to transposable or retro-transposable elements or models that appear to be single-exon retrocopies of protein-coding genes were excluded from the final set of models. Most Gnomon models with base differences with the genomic assembly introduced to correct frameshift-causing indel were labeled as pseudogenes and annotated without a CDS feature or protein product; but those with a strong unique hit to the SwissProt database or with a human ortholog were marked coding. Such models may indicate underlying defects in the assembly and should be considered lower confidence. Titles for these models are prefixed with "PREDICTED: LOW QUALITY PROTEIN". The resulting annotated assemblies and the annotated products for all 17 assemblies were loaded into RefSeq and are publicly available for download from NCBI Assembly (https://www.ncbi.nlm.nih.gov/assembly/). For each assembly, details of the evidence used for gene prediction and summary statistics of the annotations are available in the annotation reports in **Supplementary Table 20**.

Chromosome size estimate from karyotype imaging

Fibroblast cell lines were established from a female native Anna's hummingbird using enzymatic digestion on eye tissue following methods previously described[73]. The cells were accessioned in the San Diego Zoo's Frozen Zoo® (Lab# 19594). Metaphase chromosomes were harvested from the fibroblasts following Kumamoto et al.[74]. Karyotyping was done using the CytoVision Genus® System by Leica Microsystems. The karyotype represents the complete complement of a single metaphase cell. The autosomes were aligned by size and morphology with metacentric/submetacentric pairs presented first. The minute size of the microchromosomes makes it difficult to determine the exact diploid number with certainty.

The Anna's hummingbird karyotype image was then processed first into a binary representation. The rectangular area surrounding each chromosome image was then obtained from the binary representation. The relative chromosome size ratio was estimated compared to the sum of all rectangular heights. Each chromosome size estimate was obtained by multiplying this ratio to the given genome size.

The genome size was given in a diploid value, as both alleles are present in the Anna's hummingbird karyotype picture. Below is the python code used to generate the karyotype image and chromosome size estimates:

```
# Import relevant libraries and setup matplotlib
import numpy as np
from skimage.measure import regionprops
from skimage.color import rgba2rgb, rgb2gray
from scipy.ndimage import label
import matplotlib.pyplot as plt
import matplotlib.patches as mpatches
import argparse
%matplotlib inline

plt.rcParams['figure.figsize']=20,15

# genome size in bases - if image shows multiple alleles, then the size has to be adapted
accordingly
gs = 2119374518

# be sure to remove anything from the picture (i.e. labels) that is not to the karyotype
path_img_in = "/Users/pippel/Documents/Calypte_anna_in.png"
path_img_out = "/Users/pippel/Documents/Calypte_anna_out.png"
path_txt_out = "/Users/pippel/Documents/Calypte_anna_out.txt"

# loading the image
img = plt.imread(path_img_in)
plt.imshow(img)

# converting to binary
threshold = 0.5     #<---------- threshold can/should be adjusted
if img.shape[2] == 4:
    gray = 1-rgb2gray(rgba2rgb(img))
else:
    gray = 1-rgb2gray(img)

binary = (gray>threshold).astype(np.int32)
plt.imshow(binary)

# creating labels
labels = label(binary)[0]

plt.imshow(labels)
```

```
# regionprobs on the label image
regions = regionprops(labels)

sizes=sum(r.area for r in regions)

fig, ax = plt.subplots(figsize=(100, 60))
ax.imshow(binary)

plt.rcParams.update({'font.size': 32})

centroid_and_area = np.zeros((len(regions), 3))
c=0
for region in regions:
    x, y = region.centroid
    area = region.area
    minr, minc, maxr, maxc = region.bbox
    # draw rectangle around objects
    rect = mpatches.Rectangle((minc, minr), maxc - minc, maxr - minr,\
     fill=False, edgecolor='red', linewidth=2 \
     plt.text(minc, minr,                     \
     str(np.round(gs*region.area/sizes.sum()/1000000,2)), color='red')
    centroid_and_area[c] = (x, y, gs*region.area/sizes.sum()/1000000)
    c+=1
    ax.add_patch(rect)

# save image to image output file
plt.savefig(path_img_out)

# save centroid and size estimate to text file
np.savetxt(path_txt_out, centroid_and_area, fmt='%1.3f %1.3f %1.3fM')
```

Weighted read length distributions

All read lengths or molecule lengths were collected for PacBio CLR and Bionano optical maps over 1kb. The weighted read length distribution was calculated using the read length normalized by the total bases sequenced in reads of that length.

Pacbio CLR read length distribution: Read length was extracted for all subreads with Samtools[14] 1.9-1.10 `faidx` and filtered for reads over 1kb. Total bases were counted at the end to weight the bases in the read length of X.

```
# Collect read length for each subreads
samtools faidx $subreads.fasta

# length collected per genome at the end
cat *.fasta.fai | awk '{print $1"\t"$2"}' > $genome.len

# Remove data < 1kb and change unit to 1kb
awk '$2>1000 {print $2/1000}' $genome.len > $genome.1kb

# Get the fraction of a read of length LEN over TOTAL_BP
TOTAL_BP=`awk -v sum=0 '{sum+=$1} END {print sum}' $genome.1kb`
awk -v TOTAL_BP=$TOTAL_BP -v PLATFORM="PacBio" \
 '{print $1"\t"($1/TOTAL_BP)"\t"PLATFORM}' $genome.1kb \
  > $dir/$genome.1kb.weighted
```

10XG molecule length distribution: 10X Genomics molecule length distribution was estimated using the `molecule_length_mean` (m) from the `summary.csv` produced with `longranger`[22] `align`. We ran `longranger align` on all curated primary assemblies (**Supplementary Table 10**) except for the Skate, which we used the longranger align output (m) from the 3rd round of polishing. For the two-lined caecilian (aRhiBiv1), the largest chromosomes were broken in two scaffolds and lifted over at the end to bypass the known indexing issue in longranger for large scaffolds. We used the m for length-weighted mean molecule length and computed the exponential distribution of the molecule length following 10X Genomics recommendation on https://support.10xgenomics.com/de-novo-assembly/software/pipelines/latest/output/moleculelen. In brief, when b = 2/m, the function f(x) = b * exp(-bx), where x is the molecule length.

Bionano raw molecule length distribution: Molecule length was extracted for all .bnx files over 1kb. Lengths were weighted by total bases. The following code was applied to Opt1 (BspQI), Opt2 (BssSI), and Opt3 (DLE1) bnx files to extract the molecule length:

```
name=`basename $file | sed 's/.bnx//g' \
       | awk -F "_" '{print $1"_"$2"_"$3}'`
cat $file | grep -v "#" | grep -v "QX" \
   | awk -v name=$name '{printf "%.0f\t%s\n", $3, name}' \
    >> $name.bnx.len

# Remove data < 1kb and change unit to 1kb
awk '$1>1000 {print $1/1000}' $bn >> $genome.1kb

# Get the fraction of a read of length LEN over TOTAK_BP, add it to the PacBio results
TOTAL_BP=`awk -v sum=0 '{sum+=$1} END {print sum}' $genome.1kb`
awk -v TOTAL_BP=$TOTAL_BP -v PLATFORM=$PLATFORM         \
  '{print $1"\t"($1/TOTAL_BP)"\t"PLATFORM}' $genome.1kb \
  >> $dir/$genome.1kb.weighted
```

Hi-C chromatin interaction length distribution: Interaction distances between any two Hi-C read pairs were collected using the curated primary assemblies (**Supplementary Table 10**) as the reference. Hi-C reads were mapped using Arima mapping pipeline (https://github.com/VGP/vgp-assembly/blob/master/pipeline/salsa/arima_mapping_pipeline.sh) as we did for SALSA[8] scaffolding. After the alignment was finished, 10 million interactions to the longest scaffold was extracted using BEDTools[75]. As with the other datatypes, the sum of all interaction distances was used to normalize each distance.

```
# Largest scaffold is $scaffold and is $len long
len=`awk -v l=0 '$2>l {l=$2} END {print l}' $genome.pri.fasta.fai`
scaffold=`awk -v len=$len '$2==len {print $1}' $genome.pri.fasta.fai`

# Collect intervals >1kb
bedtools bamtobed -bedpe -i $genome.pri.bam \
  | awk -v scaff=$scaffold '$1==scaff && $4==scaff' \
  | awk '{ if($2<$5) {start=$2;} else {start=$5;} \
     if ($3<$6) end=$6;} else { end=$3 } interval=(end-start); \
     if (interval>1000) {print (interval/1000)}}' > $dir/$genome.1kb
head -n 10000000 $genome.1kb > $genome.1kb.10M
TOTAL_BP=`awk -v sum=0 '{sum+=$1} END {print sum}' $genome.1kb.10M`
awk -v TOTAL_BP=$TOTAL_BP -v PLATFORM=$PLATFORM \
  '{print $1"\t"($1/TOTAL_BP)"\t"PLATFORM}' $genome.1kb.10M \
  > $genome.1kb.10M.weighted
```

Plotting length distribution: For 10XG linked read distances, we made a `linked.len` file which contains the genome id and the m in two columns. In R, we read the data and generate the distribution for plotting.

```
dat.10x=fread("linked.len", header=T)
result <- data.frame()
for (g in dat.10x$genome) {
  dat_genome1=dat.10x[dat.10x$genome==g,]
  dat_genome1 <- data.frame(Count=seq(from = 1, to = 100000, by = 1),\
     Length=rexp(100000, rate=2/dat_genome1$molecule_length_mean))
  totalbp=sum(as.numeric(dat_genome1$Length))
  print(totalbp)
  head(dat_genome1)
  dat_genome1$Weight=dat_genome1$y/totalbp
  dat_genome1$Platform="Linked_reads"
  dat_genome1$Genome=c(g)
  result <- rbind(result, dat_genome1)
}
```

For other platforms, the `$genome.1kb.weighted` files were concatenated with the genome id at the end in the order we want to display per genomes:

```
for genome in $(cat genome.list.srt);
do
  awk -v genome=$genome '{print $0"\t"genome}' $genome.1kb.weighted \
    >> all.1kb.weighted
done
```

Collapsed repeat calculations

Collapsed duplications were annotated using a combination of read-depth and repeat masking. The pipeline is identical to the depth calculation of SDA[76], with an updated approach to discretized copy number annotation using a hidden Markov model. Reads were mapped to each assembly using Minimap2[64] and filtering for primary alignments. In the case that a DNA fragment is sequenced by multiple subreads, only the longest aligned subread was retained. Each genome was divided into 100-base tiling windows. A window is spanned if an alignment starts within or before the window and ends in or after the window, and the read-depth is calculated per window as the number of retained reads that span the window.

The read-depth for diploid copy number is set to the average of all windows. Copy number of a collapse is calculated using a hidden Markov model that models the copy number as a hidden state with read-depth as the emitted value. Collapsed repeats were detected as contiguous spans of windows with copy number at least 4. The number of sequences missing at a collapsed duplication are represented as the range between the minimum and maximum copy number of windows in a span.

Each genome was repeat masked using a combination of RepeatMasker[77] ver. open-4.0.9 and WindowMasker[69] v1.0.0. The following repeat-libraries were used: mPhyDis1, mRhiFer1 : bats; mLynCan4 : Carnivores; mOrnAna1: monotremes; bTaeGut1, bTaeGut2, bStrHab1, bCalAnn1: birds; rGopEvg1, aRhiBiv1: Amphibia; fMasArm1, fAnaTes1, fArcCen1, fAstCal1, fCotGob3, fGouWil2, sAmbRad1: Teleost fish. Gene content was used with either NCBI annotations, or by mapping human Genbank transcripts using Minimap2[64] when no gene annotation was available (mPhyDis1, mLynCan4, mOrnAna1, aRhiBiv1).

The repeat annotation pipeline is available at:

https://github.com/ChaissonLab/SegDupAnnotation/archive/0.9.tar.gz .

BUSCO

BUSCO[30] v3.02 was run on all submitted primary assemblies (**Supplementary Table 12**) to assess gene content (C: completeness; D: Duplications; F: fragmented; M: missing), as well as on the benchmark assemblies (**Supplementary Table 13**) to assess duplications using OrthoDB v9 with vertebrata_odb9 database. Integrated software versions used were Hmmer 3.1b2, ncbi-blast-2.2.30+ and augustus-3.3. Command line used is as following:

```
run_BUSCO.py -i asm.fasta -o $out -m genome -l vertebrata_odb9
```

In addition, we performed lineage specific (-l) BUSCO runs with the closest available lineages, which uses a gene prediction model trained on human genome annotations (augustus). Specifically, we used 'laurasiatheria' for mammals, 'ave' for birds, 'tetrapoda' for Goode's thornscrub tortoise, and two-lined caecilian, 'actinopterygii' for fishes, and 'vertebrata' for the thorny skate (**Supplementary Table 12**). Command line used is as following with specific `$lineage`:

```
run_BUSCO.py -i asm.fasta -o $out -m genome -l $lineage
```

When investigating duplications, we further tried BUSCO using species specific models (-sp): 'human' for all mammals and the thorny skate; 'chicken' for all birds, Goode's thornscrub tortoise, and two-lined caecilian; and 'zebrafish' for all fishes (**Supplementary Table 12-13**). Command line used is as following with `–sp` set in addition to above example:

```
run_BUSCO.py -i asm.fasta -o $out -m genome -l $lineage -sp $species
```

We observed very similar patterns in the duplication levels (**Supplementary Table 13**), however, fluctuating slightly in the completeness score (**Supplementary Table 12**). This could reflect the quality of the gene models used for training, reference quality used to generate the initial BUSCO gene set, or the availability of the closest / identical lineage and species. With that, throughout this manuscript, we decided to use vertebrata_odb9 with the default human lineage to use the same gene set for investigating relative completeness.

Mis-joins and missed-joins in assemblies

The curated hummingbird assembly was mapped to the target assemblies with MashMap2[78] using 5 kbp segments for CLR assemblies. We used 1 kbp segments for SR assemblies to compensate for the shorter contig sizes. We ran MashMap using the following command line:

```
# For CLR assemblies
mashmap -r $ref -q $qry -t $SLURM_CPUS_PER_TASK -o $out \
  --filter_mode one-to-one --pi 95 -s 5000

# For SR assemblies
mashmap -r $ref -q $qry -t $SLURM_CPUS_PER_TASK -o $out \
  --filter_mode one-to-one --pi 95 -s 1000
```

The number of mis-joins and missed-joins were identified using a custom script available at https://github.com/jdamas13/assembly_comparison. The assembly_comparison.pl was run using the command line:

```
perl assembly_comparison.pl out.map $ref.fai $segment
```

Note that `assembly_comparison.pl` requires the more continuous assembly to be the `$qry`, and the target assembly being the `$ref` when running mashmap2. The `$segment` is adjusted accordingly to the `-s` used in MashMap.

From the summary, the number of end-to-end joins (missed-joins), rearrangements, and free-end joins were collected from the diff.summary using the following script:

```
echo -e \
"target\tmissed-joins\trearrangements\tfree-
end_breaks\tnum.scaffolds_affected_by_free-end_breaks" > summary.txt
cat $summary | awk '{print $NF}' | tr '\n' '\t' |\
 awk -v summary=$ref '{print summary"\t"$1"\t"$2"\t"$4"\t"$5}' \
 >> summary.txt
```

The number of mis-joins consist of two error types (**Supplementary Fig. 5**): 1) rearrangements and inconsistency between the curated and automated assemblies; and 2) free-end breaks where the automated assembly has a join not supported by the curated assembly. Missed-joins are contigs or scaffolds in the automated assembly that are joined in the curated assembly.

Similarly, to generate comparisons between assembly pipeline steps within a species (**Supplementary Table 14**), each intermediate assembly was mapped to its predecessor using Mashmap2 with parameters --pi 95 -s 10000.

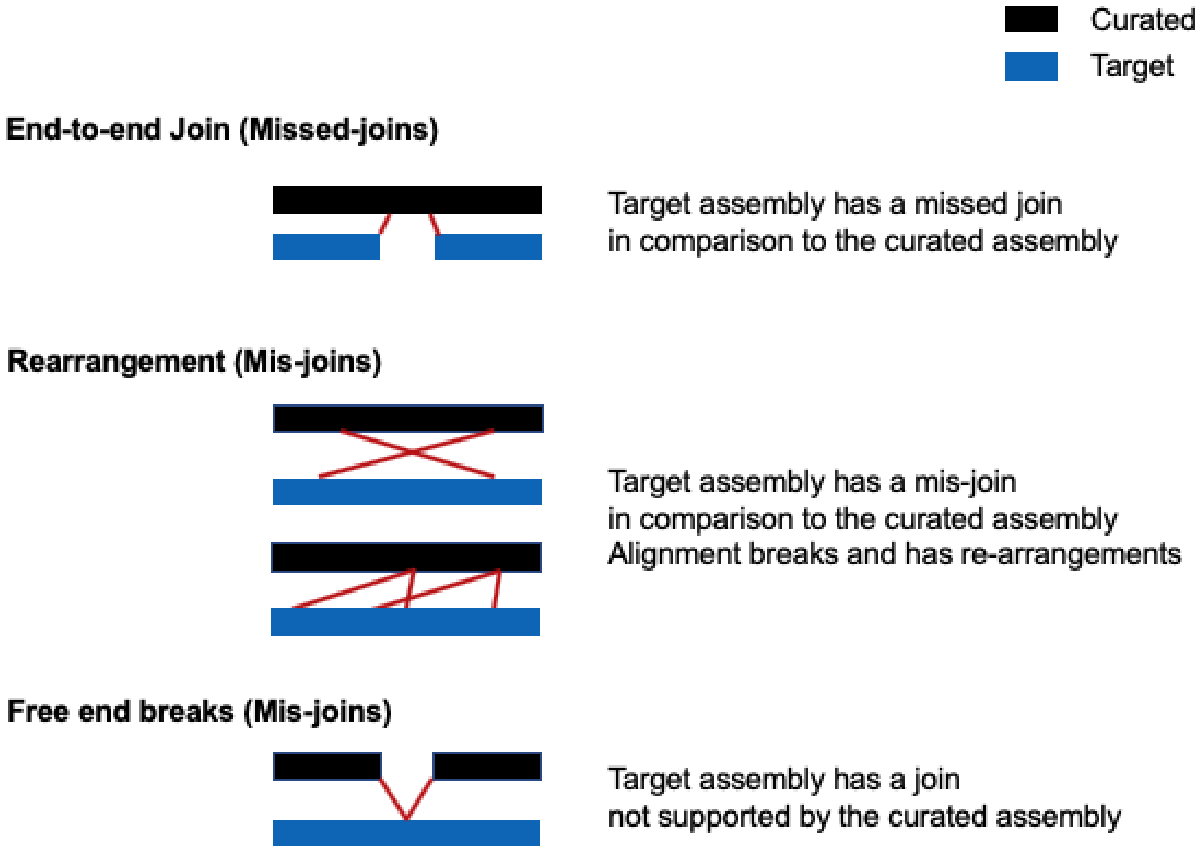

Supplementary Fig. 5 | Schematic overview of "Mis-joins" and "Missed-joins". The curated assemblies have the error fixed. Black and blue bars are contigs. Red lines indicate boundaries of the differences between the curated and the target automated assembly.

Quantifying false duplications with *k-mers*

All *21-mers* were collected from linked reads. The first 23 bp of the first read pair in 10XG reads were trimmed to remove barcode sequences. For the skate, whole genome short read sequencing data was used instead for collecting *k-mers,* as the k-mer histogram was abnormal in the linked reads. Using the k-mer histogram of the reads as a truth set for the genome, we compared *21-mers* collected from the primary and alternate assemblies.

Overlaying *k-mers* intersecting with a primary assembly in a read set is informative for inferring artificial duplications. All *k-mers* collected from single and two copies of the genome are expected to be found once in an assembly, assuming all two-copy *k-mers* are from the homozygous part of the genome with no haplotype (allele) specific duplication. A cutoff threshold was determined from the k-mer histogram of the reads as the maximum peak x 1.5 of the *k-mers* found in the assembly once. All distinct *k-mers* found more than once in the assembly within this cutoff of the k-mer counts found in reads are assembled more than expected. As an example, *k-mers* found in the assembly once peaked at 57x (**Supplementary Fig. 6**). The cutoff is therefore 86 (57 x 1.5). All *k-mers* found twice (blue), three (green), four (purple), or more (orange) times under 86x are considered and counted as falsely duplicated *k-mers*. Barcode trimming, *k-mer* counting, copy number spectrum, and false duplication counting was all performed with Merqury[37] `spectra-cn`.

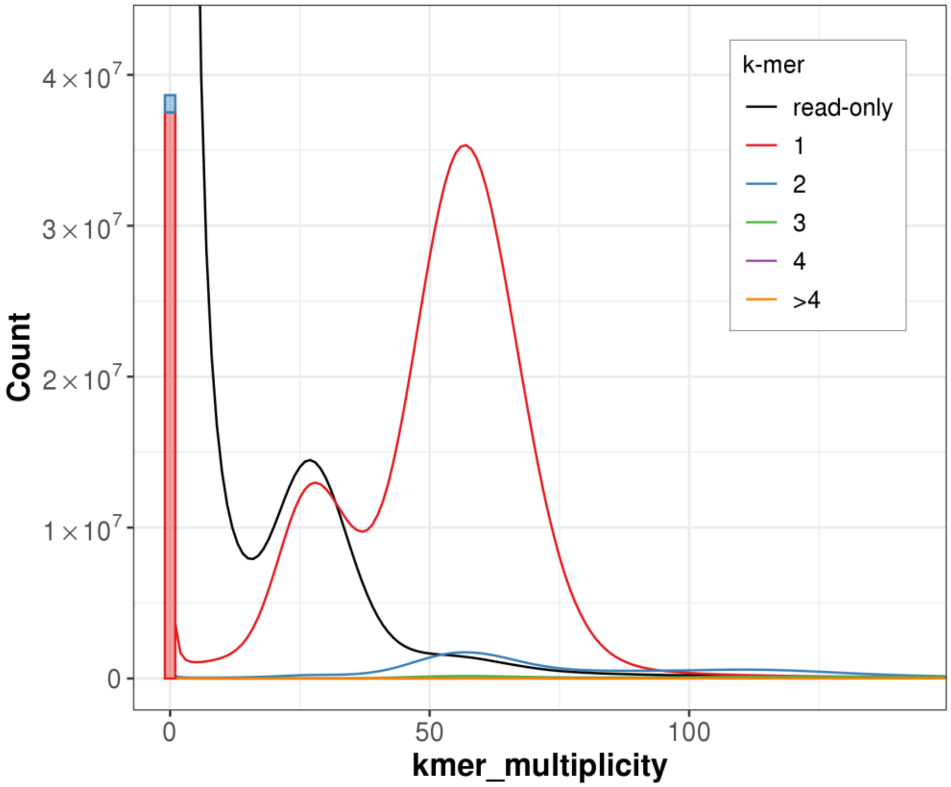

Supplementary Fig. 6 | Example histogram of the k-mer counts.

Once the *k-mers* were collected, the histogram (e.g. **Supplementary Fig. 6**) was prepared with spectra-cn of the Merqury code and shown in **Supplementary Fig. 2**:

https://github.com/marbl/merqury/blob/master/eval/spectra-cn.sh

Which generates histogram of the primary assembly in the following format:

```
Copies <tab> kmer_multiplicity <tab> Count
```

Where `Copies` are copies found in the assembly, `kmer_multiplicity` is the k-mer multiplicity found in reads, `Count` being the number of *k-mers* at each multiplicity.

The false duplication results were obtained using the following code: https://github.com/marbl/merqury/blob/master/eval/false_duplications.sh

```
cutoff=`cat $hist  | awk '$1==1 {print $2"\t"$3}' | awk -v max=0 'max<$2 {max=$2; mult=$1
} END {printf "%.0f\n", mult*(1.5)}'`
one_cp=`awk -v cutoff=$cutoff '$1==1 && $2<cutoff {sum+=$NF} END {print sum}' $hist`
two_cp=`awk -v cutoff=$cutoff '$1==2 && $2<cutoff {sum+=$NF} END {print sum}' $hist`
thr_cp=`awk -v cutoff=$cutoff '$1==3 && $2<cutoff {sum+=$NF} END {print sum}' $hist`
fou_cp=`awk -v cutoff=$cutoff '$1==4 && $2<cutoff {sum+=$NF} END {print sum}' $hist`
mor_cp=`awk -v cutoff=$cutoff '$1==">4" && $2<cutoff {sum+=$NF} END {print sum}' $hist`
DUPS_TOTAL=`echo  "$one_cp  $two_cp  $thr_cp  $fou_cp  $mor_cp"  |  awk  '{dup=$2+$3+$4+$5;
all=dup+$1} END {print $1"\t"$2"\t"$3"\t"$4"\t"$5"\t"dup"\t"all"\t"(100*dup/all)}'`
echo -e "$hist\t$DUPS_TOTAL"
```

More details on the interpretation of the k-mer histogram can be found in the KAT[36] and Merqury[37] papers.

Reliable blocks

Regions with support from CLR subreads, linked reads, raw molecule and label information (bnx) of the optical maps, and Hi-C maps from the same individual were collected. Low or high coverage regions were excluded, which are indicators of mis-assemblies. To overcome mapping biases, we required at least two independent platforms to agree for being structurally 'reliable'. For example, a repeat region longer than a CLR read may cause abnormal high coverage in CLR and linked reads from mapping biases, even if the region was well assembled locally. Longer range data such as optical maps and Hi-C interactions can complement this bias and indicate structural reliance.

Read mapping was performed individually for each platform, and coverage support information was collected on the primary assembly with Asset[35] v1.0.2 (https://github.com/dfguan/asset). Here we include a brief description of each parameter for each platform as well as codes used to generate supporting regions. A manuscript for Asset will follow with more detailed information.

Regions with not enough support (hereby "low support") were merged when less than 100 bp apart. Cutoff for defining low support differs per platform, as noted below per platforms. Reliable regions were calculated by excluding these low support regions from the assembly. Because sequencing coverage naturally drops at the end of scaffolds for optical maps and Hi-C, we included any low support region as "reliable" that overlaps 1 kbp of each ends in the scaffolds (**Supplementary Table 6**). All available optical maps were used, including those not used for hybrid scaffolding (**Supplementary Table 9**). Below are the command lines used to obtain these reliable blocks.

Gaps in the reference

Gaps and start, end of each scaffold information was obtained with `detgaps` for the primary assembly. Scaffold length was obtained with samtools. Length was then converted to region file, `asm.bed` and 1 kbp end coordinates was obtained as `asm.ends.bed` as following:

```
# Get gaps
$asset/bin/detgaps $name.fasta > gaps.bed

# Get scaffold length
samtools faidx $name.fasta

# Get scaffold region
awk '{print $1"\t0\t"$2}' $name.fasta.fai > asm.bed

# Get scaffold ends while ignore scaffolds <2kb
cat asm.bed | \
 awk '($3-$2) > 2000 {print $1"\t0\t1000\n"$1"\t"($3-1000)"\t"$3}' \
 > asm.ends.bed
```

CLR coverage

CLR reads were aligned to the primary assembly using minimap2[64] with `-x map-pb`. Once all `.paf` files were collected, `ast_pb` was run with `-M $max`, which the maximum threshold was identified from `(sequencing mean coverage) x 2.5`. The mean coverage was inferred from the estimated haploid genome size / total bases. By default, `ast_pb` only includes read alignments with a minimum of 600 bases. Where `r` is a read, `s` the starting and `e` the ending coordinate of the alignment of `r`, any read alignment with `r(s+300, e-300)` is used to avoid errors at read ends. Regions with a minimum of 10 read alignments were excluded.

Brief help message is as follows:

```
Usage: aa_pb [options] <PAF_FILE> ...
Options:
  -m INT minimum coverage [10]
  -M INT maximum coverage [400]
  -l INT bases clipped at start and end coordinates of an alignment [300]
  -h  help
```

Command lines used are as follows:

```
# Align each qry subread .fasta file to the reference index
minimap2 -x map-pb -t $cpus $ref.idx $qry > $out.paf

# Accumulate coverage and exclude low and high coverage
pafs=`ls *.paf`
max=`echo $mean_cov | awk '{printf "%.0f\n", $1*2.5}'`
$asset/bin/ast_pb -M $max $pafs > pb_M.bed"
```

Linked read coverage

The `aligned.bam` file was re-used, which was generated to obtain mapping-based QV estimates using `longranger align`. To get the $max threshold, `ast_10x` was run in two rounds. The first round was run to get the average molecule coverage. The second round was run with `-C $max`, which is (average molecule coverage) x 3.5. Note this is set higher than what was used in CLR coverage as the linked reads were aligned to both haplotypes, thus here the average molecule coverage is closer to the haploid coverage.

By default, `ast_10x` requires regions to have at least 0.15 x (average molecule coverage) or 10 molecules, whichever is higher. Molecules are only considered when the average mapping quality of the reads in it is over 20, with the inferred molecule size being longer than 1 kbp. A molecule requires shared barcodes among at least 20 reads, where any two adjacent reads are less than 20 kbp apart. The maximum number of reads in a barcode is restricted to at most 1 million; however, based on the number of reads in barcodes, most barcodes meet this filtering criteria.

Brief help message is as follows:

```
Usage: aa_10x [options] <GAP_BED> <BAM_FILEs> ...
Options:
  -x BOOL use longranger bam [False]
  -b INT  minimum number of reads for each barcode [20]
  -B INT  maximum number of reads for each barcode [1M]
  -c INT minimum molecule coverage. This or -r will be used, whichever is higher. [10]
  -r FLOAT minimum coverage ratio to the average coverage [.15]
  -C INT maximum coverage [inf]
  -q INT minimum average read mapping quality for each molecule [20]
  -l INT minimum length for a molecule [1000]
 -S INT maximum distance allowed between two adjacent reads with identical barcode to
be grouped as a molecule [20000]
  -a INT minimum number of barcodes for each molecule [5]
  -h     help
```

Command lines used are as follows:

```
# First round: accumulate molecule coverage to get the mean
$asset/bin/ast_10x -x gaps.bed aligned.bam > 10x.bed

# Avg. molecule coverage and max cutoff
mean_cov=`awk '{sum+=$1*$2; total+=$2} END {printf "%.0f\n", sum/total}' TX.stat`
max=`echo $mean_cov | awk '{printf "%.0f\n", $1*3.5}'`

# Second round
$asset/bin/ast_10x -x -C $cutoff $gaps aligned.bam > 10x_C.bed
```

Optical map raw molecule (bnx) coverage

The curated primary assembly was first converted to in-silico reference cmaps for each label (Opt.1, 2, and 3) to align available bnx maps accordingly. The bnx were merged prior to alignment when multiple bnx were available from the same sequencing platform (Irys or Saphyr) and label. The bnx was aligned using `RefAligner` (Solve 3.3_10252018) from Bionano Solve[4] 3.3 using non-haplotype option of the sequencing platform (Irys or Saphyr), as we align bnx from both haplotypes to a pseudo-haplotype assembly. Molecule coverage was obtained with `ast_bion_bnx` using default options, which requires regions to have at least 10 molecule coverage or 0.5 x (average molecule coverage), whichever is higher. When multiple bnx files were used, all molecule coverage was gathered using the `union` function of Asset.

Brief help message is as follows:

```
Usage: ast_bion_bnx [options] <REF_CMAP> <QUERY_CMAP> <XMAP> <KEY_FN>
Options:
  -m INT   minimum molecule coverage [10]
  -M INT   maximum molecule coverage [inf]
  -r INT   minimum coverage ratio to mean coverage [.5]
  -s FLOAT minimum alignment confidence [0.0]
  -O STR   output directory [.]
  -h       help
```

Command lines used are as follows:

```
# Convert primary reference assembly fasta to cmap
perl $solve_dir/HybridScaffold/10252018/scripts/fa2cmap_multi_color.pl -e $enzyme 1 -i
$ref -o $output_dir/fa2cmap

# Merge if multiple bnx are available from the same platform and label, prefix is for
example mLynCan4_Saphyr_BspQI
$tools/bionano/Solve3.3_10252018/RefAligner/7915.7989rel/RefAligner -if $prefix.list -
merge -o $prefix -bnx -stdout -stderr

# Align bnx to the reference cmap
python   $solve_dir/Pipeline/10252018/align_bnx_to_cmap.py   --prefix   $enzyme   --mol
$query_map --ref $ref_cmap --ra $solve_dir/RefAligner/7915.7989rel/ --nthreads $cpus -
-output                          $output_dir/align                          --optArgs
$solve_dir/RefAligner/7915.7989rel/optArguments_nonhaplotype_"$platform".xml        --
pipeline $solve_dir/Pipeline/10252018/

# Convert to support regions of this .bnx.
# $rmap_fn, $qmap_fn, $xmap_fn, and $key_fn are the output files
# of the above fa2cmap
$asset/bin/ast_bion_bnx $rmap_fn $qmap_fn $xmap_fn $key_fn \
  > $output_dir/bionano_"$tech"_"$enzyme".bed \
  2>ast_bion_bnx_"$tech"_"$enzyme".log

# Merge support regions when multiple enzymes were used
$asset/bin/union bnx_*/bionano_*.bed > bn.bed
```

Hi-C interaction coverage

The `$genome.pri.bam` was re-used which was generated to plot the weighted length distribution of the Hi-C interactions. Coverage information was obtained using `ast_hic` with default options, which excludes regions with less than seven interactions. An interaction is inferred from the distance of a read pair, using the starting coordinates of each read while excluding N-base gaps. Only interactions less than 15 kbp were considered in coverage to avoid noisy long-range interactions for inferring structural reliability.

Brief help message is as follows:

```
Usage: aa_hic [options] <GAP_BED> <BAM_FILEs>
Options:
  -c INT minimum coverage [7]
  -C INT maximum coverage [inf]
  -q INT minimum alignment quality [0]
  -L INT maximum insertion length, gap excluded [15000]
  -h     help
```

Command lines used is as follows:

```
# Convert alignments to support information
$asset/bin/ast_hic gaps.bed *.bam > hic.bed
```

Merging supportive regions

Once all the support information for each platform is generated, low and high coverage regions are merged and good supporting regions of each platform are obtained using BEDTools[75] 2.92.2 with the following command lines:

```
# Get too-low and too-high coverage low support regions by merging blocks less than 100bp away
bedtools subtract -a asm.bed -b ${platform}.bed | bedtools merge -d 100 -i - > $platform.low_high.bed

# Trim off regions overlapping 1kb
bedtools subtract -a $platform.low_high.bed -b asm.ends.bed -A > $platform.low_high.trim1k.bed

# Get supporting region, using the low_high.bed to exclude <100bp blocks in between other blocks
bedtools subtract -a asm.bed -b $platform.low_high.bed > $platform.support.bed
```

In the last step, we accumulate the supporting evidence of all platforms and obtain supporting regions where >= 2 platforms agree using the following:

```
# Accumulate supports
$asset/bin/acc gaps.bed */*.support.bed > acc.bed 2> acc.log

# Merge to get reliable blocks
awk '$4>1' acc.bed | bedtools merge -i - > acc.gt2.mrg.bed

# Get low support regions by merging blocks <100bp apart
bedtools subtract -a asm.bed -b acc.gt2.mrg.bed | bedtools merge -d 100 -i - > low_support.bed

# Get the final support region as reliable blocks
bedtools subtract -a asm.bed -b low_support.bed > reliable.bed

# Exclude low supports in <1kb scaffold boundaries for excluding end-scaffold effects
bedtools subtract -A -a low_support.bed -b asm.ends.bed > low_support.trim1k.bed
```

The scripts used here are available on:
https://github.com/VGP/vgp-assembly/tree/master/pipeline/asset.

Telomere motifs

Telomeric *6-mer* motif AATCCC and its reverse complement TTAGGG were searched in the curated assemblies using a custom script. Once the motif sites were collected, regions of enriched telomeric motif signals in 1 kbp windows were collected over threshold *S*, corrected with the *k-mer* survival rate. The corrected threshold becomes *S* x *identity^k*, where *identity* is the approximate base accuracy, which we set to 99.9%. Because the spacing of enriched telomeric motifs varies across species and assembly quality, we collected windows using various thresholds from 10% to 25%. Windows were merged when they were closer than 100 bp in chromosome-assigned scaffolds, and reported in **Supplementary Table 7**. Number of windows within the beginning or ending scaffold coordinates were collected using BEDTools[75]. Scaffold ends with windows found at a 15% threshold within 1 kbp end coordinates are reported in **Supplementary Table 6**. The following code was used to generate the data:

```
# Find telomere motifs, outputs pri.telomere
$VGP_PIPELINE/telomere/find_telomere.sh pri.fasta

# Find windows using variable $thresholds
java -cp $VGP_PIPELINE/telomere/telomere.jar FindTelomereWindows pri.telomere 99.9
$threshold > pri.windows.$threshold

# Merge telomere windows when 100bp apart
cat pri.windows.$threshold | awk '{print $2"\t"$(NF-2)"\t"$(NF-1)}' | sed 's/>//g' |
bedtools merge -d 100  > pri.windows.$threshold.bed

# Get scaffold ends with variable $ends
cat pri.lens | awk -v ends=$ends '{if ($2>(ends*2)) {print $1"\t0\t"ends"\n"$1"\t"($NF-
ends)"\t"$NF} else {print $1"\t0\t"$NF}}' > asm.ends.bed

# Get windows intersecting ends
bedtools intersect -wa -a pri.windows.$threshold.bed -b asm.ends.bed >
pri.windows.$threshold.$ends.ends.bed

# Get unique scaffold ends with a window
bedtools intersect -u -a asm.ends.bed -b pri.windows.$threshold.bed >>
pri.windows.$threshold.$ends.ends.u.bed
```

The full telomere motif finding script used is available on:

https://github.com/VGP/vgp-assembly/tree/master/pipeline/telomere/

Base pair accuracy (QV) estimate

We generated base level accuracy estimates (QVs) from the widely used mapping based approach as well as the newly developed k-mer based approach (**Extended Data Table 1** and **Supplementary Table 17**).

Mapping based approach

Longranger[22] v2.2.2 was used for generating reference index and alignments. The reference index was generated on the combined primary and alternate assemblies with `longranger mkref`, and 10XG linked reads were aligned with `longranger align`.

```
longranger-2.2.2/longranger mkref $ref.fasta
longranger-2.2.2/longranger align \
--id=$genome \
--fastq=/data/rhiea/genome10k/$genome/genomic_data/10x/ \
--sample=$genome \
--reference=refdata-$ref \
--jobmode=slurm \
--maxjobs=500 \
--jobinterval=5000 \
--disable-ui \
--nopreflight
```

For reference assembly size larger than 4G, the following memory options were applied as Longranger was tuned for human genomes with `--override=$pipeline/longranger/override_4G.json` option.

The `override_4G.json` looks as following:

```
{
"ALIGNER_CS.ALIGNER._LINKED_READS_ALIGNER.BARCODE_AWARE_ALIGNER": { "chunk.mem_gb": 48
},
"ALIGNER_CS.ALIGNER._LINKED_READS_ALIGNER.MERGE_POS_BAM": { "join.mem_gb": 48 },
"ALIGNER_CS.ALIGNER._REPORTER.FILTER_BARCODES": { "join.mem_gb": 48 },
"ALIGNER_CS.ALIGNER._REPORTER.REPORT_LENGTH_MASS": { "chunk.mem_gb": 32 }
}
```

From the summary.csv that longranger align outputs, the mean coverage was obtained. Then, variants were called with freebayes[13] 1.3.1 from the possorted_bam.bam file, which are indicative of base-pair errors as we align reads from the same individual. This is the same step used for finding target bases to polish. We use --skip-coverage (mean_cov*12) to avoid variant calls in regions with excessive coverage depth, as the mapping results in this region is not reliable for variant calling. Basic filtering was applied on the called variants, to filter out (1) low quality (>1) sites, (2) select target sites called as homozygous-like variants (all reads support base change to one allele), and (3) heterozygous-like variant calls when both suggestive alleles do not match the reference. In the latter case, the longest allele was chosen.

(https://github.com/VGP/vgp-assembly/tree/master/pipeline/freebayes-polish)

```
# Variant call
freebayes --bam $bam --skip-coverage $((mean_cov*12)) -f $fasta | bcftools view --no-
version -Ou > bcf/$.bcf

# Filtering
bcftools view -i 'QUAL>1 && (GT="AA" || GT="Aa")' -Oz --threads=$threads $sample.bcf >
$sample.changes.vcf.gz
```

```
# Collect number of bases affected (NUM_VAR)
bcftools view -H -Ov $genome.changes.vcf.gz | awk -F "\t" '{print $4"\t"$5}' | awk '{lenA=length($1); lenB=length($2); if (lenA < lenB ) {sum+=lenB-lenA} else if ( lenA > lenB ) { sum+=lenA-lenB } else {sum+=lenA}} END {print sum}' > $genome.numvar
NUM_VAR=`cat $genome.numvar`
echo "Total num. bases subject to change: $NUM_VAR"
```

Mappable region was obtained by excluding low (<3x) and high (mean_cov x 12) coverage. (https://github.com/VGP/vgp-assembly/tree/master/pipeline/qv)

```
# Num. of bases in mappable region (NUM_BP)
l_filter=3
h_filter=$((mean_cov*12))
samtools view -F 0x100 -u $bam | bedtools genomecov -ibam - -split > aligned.genomecov
awk -v l=$l_filter -v h=$h_filter '{if ($1=="genome" && $2>l && $2<h) {numbp += $3}} END {print numbp}' aligned.genomecov > $genome.numbp
NUM_BP=`cat $genome.numbp`

# QV calculation
QV=`echo "$NUM_VAR $NUM_BP" | awk '{print (-10*log($1/$2)/log(10))}'`
echo "QV of this genome $genome: $QV"
```

Number of bases affected (NUM_VAR) and bases in mappable regions (NUM_BP) are obtained per primary and alternate assemblies at the end, according to the reference sequence name.

K-mer based approach

Similarly to the mapping based approach, the number of *k-mers* associated with base errors (similar to NUM_VAR in mapping based approach) and total number of *k-mers* in the assembly (NUM_BP, respectively) are obtained and used for QV calculation. This assumes all *k-mers* occurring in the genome are observed in the short reads, and any *k-mer*s not found in the short reads but in the assembly is considered to have originated from base error(s). More details regarding implementation is described in the Merqury paper[37]. This approach is independent from mapping, and is able to estimate QV across all assembled bases.

Once the *k-mers* were obtained as described in the **Quantifying false duplications with k-mers** section, Merqury `spectra-cn` was run using the following commands:

```
merqury.sh $read.meryl $pri.fasta $alt.fasta $out
```

Which generates spectra-asm and spectra-cn histograms shown in **Supplementary Fig. 2** as well as QV stats.

RNA-seq and ATAC-seq Mappability

RNAseq data from 44 zebra finch brain tissues (11 distinct regions, 4 adult male individuals) were trimmed for adaptors using fastq-mcf as part of the ea-utils package[79] 1.05 and mapped to the Sanger (TaeGut3.2.4) and VGP (bTaeGut1) genomic assemblies using STAR[80] v2.7.1a with default options. Reads were considered uniquely mapped if they matched only one location in the assembly, while multi-mapped (<20) or mapped to many (>20) were also counted. Total mapped was a summary of these categories. Mapping reports were summarized using MultiQC[81] v1.7 and compiled in R for significance testing. The following command lines were used:

```
STAR
--runMode alignReads \
--runThreadN 8 \
--genomeDir ${REFERENCE} \
--readFilesIn                                 ${TRIMDIR}/${SAMPLE}_R1_trimmed.fastq.gz
${TRIMDIR}/${SAMPLE}_R2_trimmed.fastq.gz \
--readFilesCommand zcat \
--outFileNamePrefix ${OUTPUT}/vgp_${SAMPLE} \
--outSAMtype BAM SortedByCoordinate
```

ATAC-seq libraries from 12 zebra finch brain tissues (4 distinct regions, 3 male birds per region) were prepared using the Omni-ATAC-seq method (https://protocolexchange.researchsquare.com/article/nprot-6107/v1) and were sequenced on the Illumina NextSeq 500 with 75 bp paired end reads. The reads were then trimmed with Trim Galore[82] v0.6.5. Next, the reads were aligned to each assembly using Bowtie2[83] v2.4.1. The average of each mapping statistic summary log (mapped zero times, mapped exactly 1 time and mapped multiple times) were calculated for each assembly. The following command lines were used:

```
# Trimming adapters
trim_galore --paired --nextera myRead_R1.fastq myRead_R2.fastq

# Assembly alignment
bowtie2   -x   genome_assembly.fa   --sensitive   -1   my_reads_trimmed_1.fq.gz   -2
my_reads_trimmed_2.fq.gz -S my_reads_trimmed.sam 2> my_reads_trimmed.log
```

Mapping statistics for both RNA-seq and ATAC-seq on each assembly were tested for significant difference in means using a paired two sample t-test (alpha=0.05).

False gene annotation in previous assemblies

We detected evidence of erroneous coding sequences in previous assemblies of the zebra finch, platypus, and climbing perch for the genes which are related to specific complex traits[84,85] or, included in the BUSCO gene set[30]. To identify the erroneous annotations, such as false duplications or truncated sequences due to missamblies, we collected exon sequences from the VGP annotation of the genes and performed blastn v2.6.0+ searches[86] against both the previous and VGP assembly, with options -task blastn, -perc_identity 90, and -evalue 0.00001 (**Supplementary table 21**). Among the hits found from the blast search, we defined false duplications of an exon when duplicated hits within the same scaffold were found on the previous assembly only. Also, we detected truncated exons, where the length of the blast hit was shorter than the length of query exon. For visualization, we used Gene Structure Display Server v2.0+[87] and manually modified the display in order to handle small discrepancies between elements. For the intuitive visualization of platypus' vitellogenin-2 gene, we visualized only the scaffolds with more than three blast hits of the previous assembly.

GC-content and missing sequences in prior assemblies

We investigated GC-content of protein coding genes and flanking sequences of 17 VGP genomes (mLynCan4, mPhyDis1, mOrnAna1, mRhiFer1, bStrHab1, bTaeGut2, bCalAnn1, bTaeGut1, aRhiBiv1, rGopEvg1, fArcCen1, fCotGob3.1, fMasArm1.2, fAnaTes1.2, fAstCal1.2, fGouWil2.1, and sAmbRad1). All assemblies and annotation files were downloaded from NCBI RefSeq[88]. We manually annotated UTR exons at the start or end of each transcript that did not overlap with CDS regions. For each gene, the longest transcript was selected as a representative transcript. We excluded genes located within the 30kbp of the end of scaffolds, genes with less than four coding exons, or the genes flagged as "partial" from our downstream analysis. Introns more than 25 bp were classified according to their position: 5' or 3'UTR intron - in 5' or 3'UTR, first intron; between the first and next coding exon, internal intron; between internal coding exons, last intron; and between the internal and last coding exon. After excluding coding exons under 10bp, we calculated average GC-content of non-overlapping 100bp windows in the upstream and downstream 30 kbp regions and of the UTRs, coding exons, introns for each species with BEDtools[75] nuc (v2.26).

Genome alignments were made among the VGP primary, VGP alternate, and prior assemblies with Cactus[89] for zebra finch, Anna's hummingbird, platypus, and climbing perch[90,91] in order to estimate the ratio of previously missing sequences. We extracted VGP genomic regions that were not aligned with the prior assemblies by using halLiftover[92] and BEDtools[75] subtract. Ratio of missing sequences was estimated for the UTRs, exons, introns and non-overlapping 100bp windows in the flanking 2 kbp upstream and downstream sequences by calculating the ratio of overlaps with the regions that were not aligned with the prior assemblies, with BEDtools[43] intersect and groupby.

We investigated zebra finch genes whose 5' upstream sequences were previously missing. We downloaded NCBI remap alignment (https://www.ncbi.nlm.nih.gov/genome/tools/remap) between the VGP (bTaeGut1_v1.p) and prior assembly (Taeniopygia_guttata-3.2.4). The VGP genomic regions that were not already mapped to the prior assembly were extracted by BEDtools[75] subtract. The CpG island prediction result of unmasked VGP zebra finch assembly was downloaded from UCSC genome browser. We overlapped coordinates of unmapped regions, +-2kbp upstream region of each gene, CpG islands, and ATAC-seq peaks by using BEDtools intersect. To cross-check the absence of GC-rich upstream sequences of *DRD1B* and *ER81* in the prior assembly, we performed BlastN[86] against the VGP and prior assembly using the following parameters: *-task blastn -dust no -evalue 0.000001* and confirmed that no hits were found against the prior assembly. We remapped the coordinates of previous *DRD1B* and *ER81* transcripts to the VGP assembly by NCBI Remap and confirmed that the structure of previous genes were disrupted by the missing sequences. BEDtools makewindows and nuc were used to calculate GC-content in non-overlapping windows of 100 bp size across the VGP assembly and visualized in integrative genomics viewer[93].

Chromosome evolution analyses

As the species divergence were too high to generate a complete genome-to-genome alignment, we estimated chromosome orthology between species by using BUSCO[30] genes. We used the BUSCO gene annotations generated using the vertebrata_odb9 database for the 16 VGP species (mLynCan4, mRhiFer1, mPhyDis1, mOrnAna1, bCalAnn1, bTaeGut1, bStrHab1, rGopEvg1, aRhiBiv1, fGouWil2, fAstCal1, fArcCen1, fCotGob3, fMasArm1, fAnaTes1, and sAmbRad1), and additionally performed the same BUSCO analysis on the primary assembly of the human genome reference (GRCh38.p12). We used ChrOrthLink (https://github.com/chulbioinfo/chrorthlink) to identify and visualize shared 'complete singleton BUSCO genes', which defines 1:1 orthologous chromosomal regions in all species. Among the total gene set, we identified 1,147 vertebrate BUSCO genes that were present and highly conserved as single copy in all 16 VGP species and human assemblies. The transcription start position of each gene was used to link orthologous chromosomes between different species and visualized using genoPlotR[94] v3.5.3 (**Fig. 5a**). We also calculated the average number of chromosomes that have orthologous segments between human or skate to all other lineages (**Supplementary Table 22**). All input data and scripts are available on github: https://github.com/chulbioinfo/chrorthlink.

For the analysis of chromosome evolution, we used the four mammalian genomes reported in this work (greater horseshoe bat, pale spear-nosed bat, Canada lynx, and platypus), four bat genomes from our Bat 1K companion study[20] (velvety free-tailed bat, greater mouse-eared bat, Kuhl's pipistrelle, and Egyptian fruit bat), the human genome (GRChg38.p12), and the chicken genome (galGal6a) as an outgroup for all mammals. Pairwise alignments of the chicken genome, and each VGP assembly listed above to the human genome were generated using LastZ[67] (v1.04) using the following parameters: *C = 0 E = 30 H = 2000 K = 3000 L = 2200 O = 400*. The pairwise alignments were converted into the UCSC chain and net formats with axtChain (parameters: *-minScore = 1000 -verbose = 0 -linearGap = medium* for mammals or *-linearGap = loose* for chicken) followed by chainAntiRepeat, chainSort, chainPreNet, chainNet and netSyntenic, all with default parameters[95]. The Bat 1K genomes were aligned to the human genome with LastZ using the following parameters: *K = 2400, L = 3000, Y = 9400, H = 2000*. After building chains, we applied RepeatFiller[96], which detects novel alignments between repetitive regions. After RepeatFiller, we applied chainCleaner[97] with parameters *-LRfoldThreshold= 2.5 -doPairs -LRfoldThresholdPairs = 10 -maxPairDistance = 10000 -maxSuspectScore = 100000 -minBrokenChainScore = 75000* to improve alignment specificity. Pairwise alignment chains were converted into alignment nets using a modified version of chainNet[95] that computes real scores of partial nets[97]. Next, pairwise synteny blocks were defined using maf2synteny[98] at 100, 300, and 500 kbp resolutions.

Evolutionary breakpoint regions (EBRs) were detected and classified on the basis of where on the phylogeny the breakpoint occurred using a statistical approach described in Farre et al. (2016)[99]. Using this approach, we identified, at 300 Kbp resolution of syntenic fragments, a total of 698 uniquely classified evolutionary breakpoint regions (EBRs), 80 reuse EBRs and 243 EBRs with uncertain classification. We manually curated EBRs with a unique phylogenetic classification (*N* = 698) by (a) removing EBRs that were defined as ≤1 Mbp to avoid misclassified EBRs due to lack of alignment (*N* = 61); (b) merging those EBRs that were spaced ≤150 apart to avoid alignment bias in repeat-rich regions (*N* = 47); (c) separating those EBRs re-classified as reuse in the previous step (*N* = 14); (d) excluding all breakpoints that did not span reliable blocks (as we defined in the Reliable blocks section) of the VGP genome assemblies by comparing the EBR coordinates with the computed reliable blocks (*N* = 27); and, (e) separating non-specific chiropteran EBRs by comparison to other laurasiatherian genomes (cattle, goat, pig, horse, cat, and dog; *N* = 27). The curated dataset comprised 522 EBRs. The rate of chromosome rearrangement (EBRs/My) between ancestral nodes and species was calculated using the number of EBRs detected for each phylogenetic branch divided by the estimated length of each branch (in My) of the phylogeny. Branch length was obtained from TimeTree[100]. The *t* statistic for each branch was obtained by calculating the difference between the rearrangement rate on the branch and the mean rate across all of

the branches, and then normalizing for the standard error. *P* values were corrected by false discovery rate using the p.adjust function from the R package (https://www.R-project.org).

The coordinates of each chiropteran EBR were uploaded into the UCSC Genome Browser, and all annotated genes within and immediately flanking (± 150 Kp) the boundaries of the breakpoint as defined in the human genome (RefSeq Annotation Release 109) were identified. The example genes with rearrangements that we studied in detail mapped to an orthologous positions on human chromosome 15q25.1, and a chiropteran interchromosomal EBR that mapped to an orthologous position on human chromosome 6p22.1. To verify the specificity of this *STARD5* gene rearrangement, we compared all isoforms of the STARD5 protein annotated in the genomes of the bats and human using Clustal Omega[101]. Furthermore, to investigate if the greater horseshoe bat transcripts would encode a functional protein, we used evidence from RNA sequencing, available as RNA tracks in the NCBI genome data viewer[102].

For the chiropteran interchromosomal EBR, we first improved the definition of the breakpoint boundaries by visual inspection of the pairwise whole-genome alignments. In each non-human species, the presence or absence of the 12 genes annotated within and flanking this locus in the human genome was determined by performing: (a) direct search of orthologs of the human genes in the RefSeq annotations for each of the bat species; (b) blast search of the human genes, mRNA and proteins in each of the bat species genomes, including both primary and alternate assemblies for the VGP genomes; and (c) projection of the Bat1K coding gene annotation of the greater horseshoe bat[20] to each of the other bat genomes using TOGA (Kirilenko et al, in preparation; https://github.com/hillerlab/TOGA). In brief, TOGA uses machine learning to infer orthologous genes between related species and accurately distinguish orthologs, paralogs and processed pseudogenes. TOGA takes as input pairwise genome alignment chains between a designated reference (here greater horseshoe bat) and query genome (the other five bat species), coding transcript annotations for the reference species and a file linking genes to transcripts isoforms. Then, for each gene, TOGA identifies the chain(s) that aligns the putative ortholog in the query using features capturing synteny and the amount of aligning exonic and intronic sequence; it identifies the exon/intron structure by aligning the reference gene to the orthologous query locus using CESAR[68] v2.0 in multi-exon mode. The gene annotations used for direct ortholog search were: RefSeq Annotation Release 100 for velvety free-tailed bat, greater mouse-eared bat, Kuhl's pipistrelle and greater horseshoe bat; RefSeq Annotation Release 101 for Egyptian fruit bat and pale spear-nosed bat; and, RefSeq Annotation Release 102 for Canada lynx. Genes and mRNA molecule searches were performed with NCBI discontiguous MegaBlast[86] using the following parameters: *blastn -task 'dc-megablast' -evalue '1e-50' -template_type 'coding' -template_length '18'*. Protein searches were performed using NCBI tblastn[86] with default parameters.

Supplementary References

1. Chin, C.-S. *et al.* Phased diploid genome assembly with single-molecule real-time sequencing. *Nat. Methods* **13**, 1050–1054 (2016).
2. Kronenberg, Z. N. *et al.* Extended haplotype phasing of de novo genome assemblies with FALCON-Phase. *Nat. Commun.* (2020) doi:10.1038/s41467-020-20536-y.
3. Koren, S. *et al.* De novo assembly of haplotype-resolved genomes with trio binning. *Nat. Biotechnol.* (2018) doi:10.1038/nbt.4277.
4. Bionano Genomics, Inc. Bionano Software Downloads. https://bionanogenomics.com/support/software-downloads/.
5. Durand, N. C. *et al.* Juicer Provides a One-Click System for Analyzing Loop-Resolution Hi-C Experiments. *Cell Syst.* **3**, 95–98 (2016).
6. Zhang, G. *et al.* Comparative genomics reveals insights into avian genome evolution and adaptation. *Science* **346**, 1311–1320 (2014).
7. Dudchenko, O. *et al.* De novo assembly of the Aedes aegypti genome using Hi-C yields chromosome-length scaffolds. *Science* **356**, 92–95 (2017).
8. Ghurye, J. *et al.* Integrating Hi-C links with assembly graphs for chromosome-scale assembly. *PLOS Comput. Biol.* **15**, e1007273 (2019).
9. English, A. C. *et al.* Mind the Gap: Upgrading Genomes with Pacific Biosciences RS Long-Read Sequencing Technology. *PLOS ONE* **7**, e47768 (2012).
10. Bradnam, K. R. *et al.* Assemblathon 2: evaluating de novo methods of genome assembly in three vertebrate species. *GigaScience* **2**, 10 (2013).
11. Chin, C.-S. *et al.* Nonhybrid, finished microbial genome assemblies from long-read SMRT sequencing data. *Nat. Methods* **10**, 563–569 (2013).
12. Walker, B. J. *et al.* Pilon: An Integrated Tool for Comprehensive Microbial Variant Detection and Genome Assembly Improvement. *PLOS ONE* **9**, e112963 (2014).
13. Garrison, E. & Marth, G. *Haplotype-based variant detection from short-read sequencing*. http://arxiv.org/abs/1207.3907 (2012).
14. Li, H. *et al.* The Sequence Alignment/Map format and SAMtools. *Bioinformatics* **25**, 2078–2079 (2009).
15. Ning, Z. & Harry, E. Scaff10X. https://github.com/wtsi-hpag/Scaff10X.
16. Dussex, N. *et al.* Population genomics reveals the impact of long-term small population size in the critically endangered kākāpō. *Prep.* (2020).
17. Jarvis, E. D. Evolution of vocal learning and spoken language. *Science* **366**, 50–54 (2019).
18. Vernes, S. C. & Wilkinson, G. S. Behaviour, biology and evolution of vocal learning in bats. *Philos. Trans. R. Soc. B Biol. Sci.* **375**, 20190061 (2020).
19. Warren, W. C. *et al.* Genome analysis of the platypus reveals unique signatures of evolution. *Nature* **453**, 175–183 (2008).
20. Jebb, D. *et al.* Six reference-quality genomes reveal evolution of bat adaptations. *Nature* **583**, 578–584 (2020).
21. Roach, M. J., Schmidt, S. A. & Borneman, A. R. Purge Haplotigs: allelic contig reassignment for third-gen diploid genome assemblies. *BMC Bioinformatics* **19**, 460 (2018).
22. Bishara, A. *et al.* Read clouds uncover variation in complex regions of the human genome. *Genome Res.* **25**, 1570–1580 (2015).
23. Li, H. Minimap and miniasm: fast mapping and de novo assembly for noisy long sequences. *Bioinformatics* **32**, 2103–2110 (2016).
24. Li, H. Aligning sequence reads, clone sequences and assembly contigs with BWA-MEM. *ArXiv13033997 Q-Bio* (2013).
25. Ruan, J. & Li, H. Fast and accurate long-read assembly with wtdbg2. *Nat. Methods* **17**, 155–158 (2020).
26. Koren, S. *et al.* Canu: scalable and accurate long-read assembly via adaptive k-mer weighting and repeat separation. *Genome Res.* gr.215087.116 (2017) doi:10.1101/gr.215087.116.
27. Chow, W. *et al.* gEVAL - a web-based browser for evaluating genome assemblies. *Bioinforma. Oxf.*

Engl. **32**, 2508–2510 (2016).
28. O'Quin, C. T., Drilea, A. C., Conte, M. A. & Kocher, T. D. Mapping of pigmentation QTL on an anchored genome assembly of the cichlid fish, Metriaclima zebra. *BMC Genomics* **14**, 287 (2013).
29. Albertson, R. C. *et al.* Genetic basis of continuous variation in the levels and modular inheritance of pigmentation in cichlid fishes. *Mol. Ecol.* **23**, 5135–5150 (2014).
30. Waterhouse, R. M. *et al.* BUSCO Applications from Quality Assessments to Gene Prediction and Phylogenomics. *Mol. Biol. Evol.* **35**, 543–548 (2018).
31. Guan, D. *et al.* Identifying and removing haplotypic duplication in primary genome assemblies. *Bioinformatics* **36**, 2896–2898 (2020).
32. Yunis, J. J. & Hoffman, W. R. Nuclear Enzymes, Fragile Sites, and Cancer. *J. Gerontol.* **44**, 37–44 (1989).
33. Earl, D. *et al.* Assemblathon 1: a competitive assessment of de novo short read assembly methods. *Genome Res.* **21**, 2224–2241 (2011).
34. Vurture, G. W. *et al.* GenomeScope: fast reference-free genome profiling from short reads. *Bioinformatics* **33**, 2202–2204 (2017).
35. Guan, D. Asset. https://github.com/dfguan/asset.
36. Mapleson, D., Garcia Accinelli, G., Kettleborough, G., Wright, J. & Clavijo, B. J. KAT: a K-mer analysis toolkit to quality control NGS datasets and genome assemblies. *Bioinformatics* **33**, 574–576 (2017).
37. Rhie, A., Walenz, B. P., Koren, S. & Phillippy, A. M. Merqury: reference-free quality, completeness, and phasing assessment for genome assemblies. *Genome Biol.* **21**, 245 (2020).
38. Howe, K. *et al.* Significantly improving the quality of genome assemblies through curation. *GigaScience* **10**, (2021).
39. Ghareghani, M. *et al.* Strand-seq enables reliable separation of long reads by chromosome via expectation maximization. *Bioinformatics* **34**, i115–i123 (2018).
40. Garg, S. *et al.* Chromosome-scale, haplotype-resolved assembly of human genomes. *Nat. Biotechnol.* 1–4 (2020) doi:10.1038/s41587-020-0711-0.
41. Korlach, J. *et al.* De novo PacBio long-read and phased avian genome assemblies correct and add to reference genes generated with intermediate and short reads. *GigaScience* **6**, 1–16 (2017).
42. Lewin, H. A., Graves, J. A. M., Ryder, O. A., Graphodatsky, A. S. & O'Brien, S. J. Precision nomenclature for the new genomics. *GigaScience* **8**, (2019).
43. Lewin, H. A. *et al.* Earth BioGenome Project: Sequencing life for the future of life. *Proc. Natl. Acad. Sci. U. S. A.* **115**, 4325–4333 (2018).
44. Koepfli, K.-P., Paten, B., Genome 10K Community of Scientists & O'Brien, S. J. The Genome 10K Project: a way forward. *Annu. Rev. Anim. Biosci.* **3**, 57–111 (2015).
45. Zhang, G. *et al.* Genomics: Bird sequencing project takes off. *Nature* **522**, 34 (2015).
46. Teeling, E. C. *et al.* Bat Biology, Genomes, and the Bat1K Project: To Generate Chromosome-Level Genomes for All Living Bat Species. *Annu. Rev. Anim. Biosci.* **6**, 23–46 (2018).
47. Jarvis, E. D. *et al.* Whole-genome analyses resolve early branches in the tree of life of modern birds. *Science* **346**, 1320–1331 (2014).
48. Meredith, R. W. *et al.* Impacts of the Cretaceous Terrestrial Revolution and KPg extinction on mammal diversification. *Science* **334**, 521–524 (2011).
49. A reference standard for genome biology. *Nat. Biotechnol.* **36**, 1121–1121 (2018).
50. Jarvis, E. D. Perspectives from the Avian Phylogenomics Project: Questions that Can Be Answered with Sequencing All Genomes of a Vertebrate Class. *Annu. Rev. Anim. Biosci.* **4**, 45–59 (2016).
51. Boomsma, J. J. Forum The Global Ant Genomics Alliance (GAGA). 7 (2017).
52. Lopez, J. V., Kamel, B., Medina, M., Collins, T. & Baums, I. B. Multiple Facets of Marine Invertebrate Conservation Genomics. *Annu. Rev. Anim. Biosci.* **7**, 473–497 (2019).
53. Chen, Z. *et al.* Ultra-low input single tube linked-read library method enables short-read second-generation sequencing systems to generate highly accurate and economical long-range sequencing information routinely. *Genome Res.* gr.260380.119 (2020) doi:10.1101/gr.260380.119.
54. Wang, O. *et al.* Efficient and unique cobarcoding of second-generation sequencing reads from long DNA molecules enabling cost-effective and accurate sequencing, haplotyping, and de novo assembly.

Genome Res. **29**, 798–808 (2019).
55. Zhang, F. *et al.* Haplotype phasing of whole human genomes using bead-based barcode partitioning in a single tube. *Nat. Biotechnol.* **35**, 852–857 (2017).
56. Jain, M. *et al.* Nanopore sequencing and assembly of a human genome with ultra-long reads. *Nat. Biotechnol.* **36**, 338–345 (2018).
57. Shafin, K. *et al.* Nanopore sequencing and the Shasta toolkit enable efficient de novo assembly of eleven human genomes. *Nat. Biotechnol.* 1–10 (2020) doi:10.1038/s41587-020-0503-6.
58. Wenger, A. M. *et al.* Accurate circular consensus long-read sequencing improves variant detection and assembly of a human genome. *Nat. Biotechnol.* **37**, 1155–1162 (2019).
59. Ondov, B. D. *et al.* Mash: fast genome and metagenome distance estimation using MinHash. *Genome Biol.* **17**, 132 (2016).
60. Aken, B. L. *et al.* The Ensembl gene annotation system. *Database J. Biol. Databases Curation* **2016**, (2016).
61. UniProt Consortium. UniProt: a worldwide hub of protein knowledge. *Nucleic Acids Res.* **47**, D506–D515 (2019).
62. Frankish, A. *et al.* GENCODE reference annotation for the human and mouse genomes. *Nucleic Acids Res.* **47**, D766–D773 (2019).
63. Slater, G. S. C. & Birney, E. Automated generation of heuristics for biological sequence comparison. *BMC Bioinformatics* **6**, 31 (2005).
64. Li, H. Minimap2: pairwise alignment for nucleotide sequences. *Bioinforma. Oxf. Engl.* **34**, 3094–3100 (2018).
65. Altschul, S. F., Gish, W., Miller, W., Myers, E. W. & Lipman, D. J. Basic local alignment search tool. *J. Mol. Biol.* **215**, 403–410 (1990).
66. She, R. *et al.* genBlastG: using BLAST searches to build homologous gene models. *Bioinforma. Oxf. Engl.* **27**, 2141–2143 (2011).
67. Harris, R. S. Improved Pairwise Alignment of Genomic DNA. (2007).
68. Sharma, V., Schwede, P. & Hiller, M. CESAR 2.0 substantially improves speed and accuracy of comparative gene annotation. *Bioinforma. Oxf. Engl.* **33**, 3985–3987 (2017).
69. Morgulis, A., Gertz, E. M., Schäffer, A. A. & Agarwala, R. WindowMasker: window-based masker for sequenced genomes. *Bioinforma. Oxf. Engl.* **22**, 134–141 (2006).
70. Kapustin, Y., Souvorov, A., Tatusova, T. & Lipman, D. Splign: algorithms for computing spliced alignments with identification of paralogs. *Biol. Direct* **3**, 20 (2008).
71. Lowe, T. M. & Eddy, S. R. tRNAscan-SE: a program for improved detection of transfer RNA genes in genomic sequence. *Nucleic Acids Res.* **25**, 955–964 (1997).
72. Nawrocki, E. P. *et al.* Rfam 12.0: updates to the RNA families database. *Nucleic Acids Res.* **43**, D130-137 (2015).
73. Houck, M. L., Ryder, O. A., Váhala, J., Kock, R. A. & Oosterhuis, J. E. Diploid chromosome number and chromosomal variation in the white rhinoceros (Ceratotherium simum). *J. Hered.* **85**, 30–34 (1994).
74. Kumamoto, A. T., Charter, S. J., Houck, M. L. & Frahm, M. Chromosomes of Damaliscus (Artiodactyla, Bovidae): simple and complex centric fusion rearrangements. *Chromosome Res. Int. J. Mol. Supramol. Evol. Asp. Chromosome Biol.* **4**, 614–621 (1996).
75. Quinlan, A. R. & Hall, I. M. BEDTools: a flexible suite of utilities for comparing genomic features. *Bioinformatics* **26**, 841–842 (2010).
76. Vollger, M. R. *et al.* Long-read sequence and assembly of segmental duplications. *Nat. Methods* **16**, 88–94 (2019).
77. Tarailo-Graovac, M. & Chen, N. Using RepeatMasker to identify repetitive elements in genomic sequences. *Curr. Protoc. Bioinforma.* **Chapter 4**, Unit 4.10 (2009).
78. Jain, C., Koren, S., Dilthey, A., Phillippy, A. M. & Aluru, S. A fast adaptive algorithm for computing whole-genome homology maps. *Bioinformatics* **34**, i748–i756 (2018).
79. Aronesty, E. Comparison of Sequencing Utility Programs. *Open Bioinforma. J.* **7**, (2013).
80. Dobin, A. *et al.* STAR: ultrafast universal RNA-seq aligner. *Bioinformatics* **29**, 15–21 (2013).
81. Ewels, P., Magnusson, M., Lundin, S. & Käller, M. MultiQC: summarize analysis results for multiple

tools and samples in a single report. *Bioinformatics* **32**, 3047–3048 (2016).
82. Krueger, F. Trim galore. *Wrapper Tool Cutadapt FastQC Consistently Apply Qual. Adapt. Trimming FastQ Files* **516**, 517 (2015).
83. Langmead, B. & Salzberg, S. L. Fast gapped-read alignment with Bowtie 2. *Nat. Methods* **9**, 357–359 (2012).
84. Zebra Finch Expression Brain Atlas. http://www.zebrafinchatlas.org/.
85. Robinson, R. For mammals, loss of yolk and gain of milk went hand in hand. *PLoS Biol.* **6**, e77 (2008).
86. Camacho, C. *et al.* BLAST+: architecture and applications. *BMC Bioinformatics* **10**, 421 (2009).
87. Hu, B. *et al.* GSDS 2.0: an upgraded gene feature visualization server. *Bioinforma. Oxf. Engl.* **31**, 1296–1297 (2015).
88. O'Leary, N. A. *et al.* Reference sequence (RefSeq) database at NCBI: current status, taxonomic expansion, and functional annotation. *Nucleic Acids Res.* **44**, D733-745 (2016).
89. Paten, B. *et al.* Cactus: Algorithms for genome multiple sequence alignment. *Genome Res.* **21**, 1512–1528 (2011).
90. Kim, J. *et al.* False gene and chromosome losses affected by assembly and sequence errors. *Prep.* (2021).
91. Ko, B. J. *et al.* Widespread false gene gains caused by duplication errors in genome assemblies. *Prep.* (2021).
92. Hickey, G., Paten, B., Earl, D., Zerbino, D. & Haussler, D. HAL: a hierarchical format for storing and analyzing multiple genome alignments. *Bioinforma. Oxf. Engl.* **29**, 1341–1342 (2013).
93. Robinson, J. T. *et al.* Integrative Genomics Viewer. *Nat. Biotechnol.* **29**, 24–26 (2011).
94. Guy, L., Kultima, J. R. & Andersson, S. G. E. genoPlotR: comparative gene and genome visualization in R. *Bioinforma. Oxf. Engl.* **26**, 2334–2335 (2010).
95. Kent, W. J., Baertsch, R., Hinrichs, A., Miller, W. & Haussler, D. Evolution's cauldron: duplication, deletion, and rearrangement in the mouse and human genomes. *Proc. Natl. Acad. Sci. U. S. A.* **100**, 11484–11489 (2003).
96. Osipova, E., Hecker, N. & Hiller, M. RepeatFiller newly identifies megabases of aligning repetitive sequences and improves annotations of conserved non-exonic elements. *GigaScience* **8**, (2019).
97. Suarez, H. G., Langer, B. E., Ladde, P. & Hiller, M. chainCleaner improves genome alignment specificity and sensitivity. *Bioinforma. Oxf. Engl.* **33**, 1596–1603 (2017).
98. Kolmogorov, M., Raney, B., Paten, B. & Pham, S. Ragout-a reference-assisted assembly tool for bacterial genomes. *Bioinforma. Oxf. Engl.* **30**, i302-309 (2014).
99. Farré, M. *et al.* Novel Insights into Chromosome Evolution in Birds, Archosaurs, and Reptiles. *Genome Biol. Evol.* **8**, 2442–2451 (2016).
100. Kumar, S., Stecher, G., Suleski, M. & Hedges, S. B. TimeTree: A Resource for Timelines, Timetrees, and Divergence Times. *Mol. Biol. Evol.* **34**, 1812–1819 (2017).
101. Sievers, F. & Higgins, D. G. Clustal Omega for making accurate alignments of many protein sequences. *Protein Sci. Publ. Protein Soc.* **27**, 135–145 (2018).
102. NCBI Resource Coordinators. Database resources of the National Center for Biotechnology Information. *Nucleic Acids Res.* **46**, D8–D13 (2018).
