# Supplementary material for: Towards complete and error-free genome assemblies of all vertebrate species
Source: Nature. 2021 Apr 28;592(7856):737–46. doi: 10.1038/s41586-021-03451-0 (PMC8081667; doi:10.1038/s41586-021-03451-0)
Supplement: Supplementary file 2 — Reporting Summary [file 41586_2021_3451_MOESM2_ESM.pdf]

nature research

Corresponding author(s): Erich D. Jarvis, Arang Rhie, Kerstin Howe, Eugene Myers, Richard Durbin, Adam Phillippy

Last updated by author(s): Jan 8, 2021

Reporting Summary

Nature Research wishes to improve the reproducibility of the work that we publish. This form provides structure for consistency and transparency in reporting. For further information on Nature Research policies, see our Editorial Policies and the Editorial Policy Checklist.

Statistics

For all statistical analyses, confirm that the following items are present in the figure legend, table legend, main text, or Methods section.

n/a	Confirmed	
☐	☒	The exact sample size (n) for each experimental group/condition, given as a discrete number and unit of measurement
☐	☒	A statement on whether measurements were taken from distinct samples or whether the same sample was measured repeatedly
☐	☒	The statistical test(s) used AND whether they are one- or two-sided<br>*Only common tests should be described solely by name; describe more complex techniques in the Methods section.*
☒	☐	A description of all covariates tested
☒	☐	A description of any assumptions or corrections, such as tests of normality and adjustment for multiple comparisons
☐	☒	A full description of the statistical parameters including central tendency (e.g. means) or other basic estimates (e.g. regression coefficient) AND variation (e.g. standard deviation) or associated estimates of uncertainty (e.g. confidence intervals)
☒	☐	For null hypothesis testing, the test statistic (e.g. F, t, r) with confidence intervals, effect sizes, degrees of freedom and P value noted<br>*Give P values as exact values whenever suitable.*
☒	☐	For Bayesian analysis, information on the choice of priors and Markov chain Monte Carlo settings
☒	☐	For hierarchical and complex designs, identification of the appropriate level for tests and full reporting of outcomes
☒	☐	Estimates of effect sizes (e.g. Cohen's d, Pearson's r), indicating how they were calculated

Our web collection on statistics for biologists contains articles on many of the points above.

Software and code

Policy information about availability of computer code

Data collection	We collected genome sequence data of various technologies from individuals representing 16 vertebrate species.
Data analysis	All software used is at at https://github.com/VGP/vgp-assembly, as of November 2020. The assembly software and versions used are listed in Supplementary Table The specific VGP assembly pipeline used is at https://github.com/VGP/vgp-assembly/tree/master/pipeline.

For manuscripts utilizing custom algorithms or software that are central to the research but not yet described in published literature, software must be made available to editors and reviewers. We strongly encourage code deposition in a community repository (e.g. GitHub). See the Nature Research guidelines for submitting code & software for further information.

Data

Policy information about availability of data

All manuscripts must include a data availability statement. This statement should provide the following information, where applicable:

- Accession codes, unique identifiers, or web links for publicly available datasets
- A list of figures that have associated raw data
- A description of any restrictions on data availability

All raw sequence and assembly data are available at Genome Ark https://vgp.github.io/genomeark/ and the NCBI BioProject page PRJNA489243 https://www.ncbi.nlm.nih.gov/bioproject/489243

Field-specific reporting

Please select the one below that is the best fit for your research. If you are not sure, read the appropriate sections before making your selection.

☒ Life sciences ☐ Behavioural & social sciences ☐ Ecological, evolutionary & environmental sciences

For a reference copy of the document with all sections, see nature.com/documents/nr-reporting-summary-flat.pdf

Life sciences study design

All studies must disclose on these points even when the disclosure is negative.

Sample size	For most analyses, we analyzed data from a sample size of n = 16 vertebrate species, and n = 17 individuals; we had two individuals for one species (the zebra finch). For the chromosomal evolution analyses, we added an additional n = 4 bat species.
Data exclusions	Only sequence data that failed quality control were excluded or repeated.
Replication	We confirmed the ability to replicate all code using multiple rounds of assembly or analyses.
Randomization	No analyses required generating randomize data sets.
Blinding	No analyses required being blind to groups

Reporting for specific materials, systems and methods

We require information from authors about some types of materials, experimental systems and methods used in many studies. Here, indicate whether each material, system or method listed is relevant to your study. If you are not sure if a list item applies to your research, read the appropriate section before selecting a response.

Materials & experimental systems

n/a	Involved in the study	
☒	☐	Antibodies
☒	☐	Eukaryotic cell lines
☒	☐	Palaeontology and archaeology
☐	☒	Animals and other organisms
☒	☐	Human research participants
☒	☐	Clinical data
☒	☐	Dual use research of concern

Methods

n/a	Involved in the study	
☒	☐	ChIP-seq
☒	☐	Flow cytometry
☒	☐	MRI-based neuroimaging

Animals and other organisms

Policy information about studies involving animals; ARRIVE guidelines recommended for reporting animal research

Laboratory animals	The care and collection of a laboratory female zebra finch sample was done under an approved IACUC protocol at the Rockefeller University. The male laboratory sample was obtained from the approval mention of the previous reference genome in Warren et al 2011 Nature.
Wild animals	Samples of the 15 other species were collected from wild animals, with approved permits of the source institutions and local governments involved. These sources, persons with the permits, geographic location, sex and relative age are listed in Supplementary Table 8 and the BioSample submissions in NCBI and ENA.
Field-collected samples	No laboratory work was conducted on field-collected animals
Ethics oversight	Rockefeller University for the zebra finch species.

Note that full information on the approval of the study protocol must also be provided in the manuscript.
